# Evolution and enrichment of CYP5035 in *Polyporales*: functionality of an understudied P450 family

**DOI:** 10.1007/s00253-021-11444-2

**Published:** 2021-08-30

**Authors:** Nico D. Fessner, David R. Nelson, Anton Glieder

**Affiliations:** 1 Institute of Molecular Biotechnology, Graz University of Technology, NAWI Graz, Petersgasse 14, 8010 Graz, Austria; 2 Department of Microbiology, Immunology and Biochemistry, University of Tennessee Health Science Center, Memphis, TN 38163 USA

**Keywords:** *P*. *arcularius*, Enzyme discovery, CYPome, CYP5035, Detoxification

## Abstract

**Abstract:**

Bioprospecting for innovative basidiomycete cytochrome P450 enzymes (P450s) is highly desirable due to the fungi’s enormous enzymatic repertoire and outstanding ability to degrade lignin and detoxify various xenobiotics. While fungal metagenomics is progressing rapidly, the biocatalytic potential of the majority of these annotated P450 sequences usually remains concealed, although functional profiling identified several P450 families with versatile substrate scopes towards various natural products. Functional knowledge about the CYP5035 family, for example, is largely insufficient. In this study, the families of the putative P450 sequences of the four white-rot fungi *Polyporus arcularius*, *Polyporus brumalis*, *Polyporus squamosus* and *Lentinus tigrinus* were assigned, and the CYPomes revealed an unusual enrichment of CYP5035, CYP5136 and CYP5150. By computational analysis of the phylogeny of the former two P450 families, the evolution of their enrichment could be traced back to the *Ganoderma* macrofungus, indicating their evolutionary benefit. In order to address the knowledge gap on CYP5035 functionality, a representative subgroup of this P450 family of *P*. *arcularius* was expressed and screened against a test set of substrates. Thereby, the multifunctional enzyme CYP5035S7 converting several plant natural product classes was discovered. Aligning CYP5035S7 to 102,000 putative P450 sequences of 36 fungal species from Joint Genome Institute-provided genomes located hundreds of further CYP5035 family members, which subfamilies were classified if possible. Exemplified by these specific enzyme analyses, this study gives valuable hints for future bioprospecting of such xenobiotic-detoxifying P450s and for the identification of their biocatalytic potential.

**Graphical abstract:**

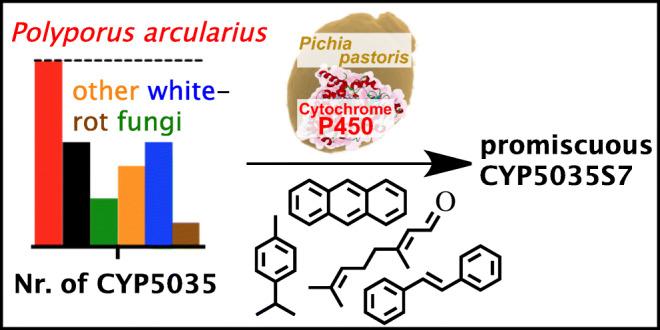

**Key points:**

• *The P450 families CYP5035 and CYP5136 are unusually enriched in P*. *arcularius.*

• *Functional screening shows CYP5035 assisting in the fungal detoxification mechanism.*

• *Some Polyporales encompass an unusually large repertoire of detoxification P450s.*

**Supplementary Information:**

The online version contains supplementary material available at 10.1007/s00253-021-11444-2.

## Introduction

The genomes of white-rot Basidiomycota code for the highest percentage of CYPome compared to the total proteome of all organisms (Chen et al. 2014). Such high density of cytochrome P450 enzymes (P450s/CYPs) helps these fungi to fully degrade the most recalcitrant aromatic polymer lignin or its low molecular weight degradation products (Peralta et al. 2017) and to survive in harsh conditions by detoxifying a vast variety of plant-based xenobiotics and other environmental hazards (Kües 2015). In order to investigate these fascinating features of fungi and their enzyme toolbox for their application in bioenergy processing or bioremediation (Yadav et al. 2019; Mäkelä et al. 2020), programmes such as the 1000 Fungal Genome project were initiated and already sequenced numerous genomes of white-rot fungi (Grigoriev et al. 2011; Grigoriev et al. 2014), providing free access to an incredible amount of P450 sequences.

Computational efforts were mobilised to annotate such enzyme sequences with unknown functions (Gerlt et al. 2011; McKay et al. 2015). With the model white-rot fungus *Phanerochaete chrysosporium* being the first (Syed and Yadav 2012), the P450s of several other wood-degrading basidiomycetes were analysed computationally (Suzuki et al. 2012; Syed et al. 2013a; Syed et al. 2014; Hori et al. 2014; Kües et al. 2015; Zhu et al. 2015; Mgbeahuruike et al. 2017) and grouped into (sub)families according to the 40% (family) and 55% (subfamily) sequence identity rules of the International P450 Nomenclature Committee (Nelson 2006). Yet, such low sequence identity, usually high selectivity of P450 enzymes and a scarce number of functionally characterised basidiomycete P450s render simulated predictions of biochemical capacities extremely difficult (Ichinose 2013). Even the closest homologues may have divergent reactivity (Gerlt 2007). Therefore, some studies attempted to express and functionally analyse the entire CYPome of *P*. *chrysosporium* and model brown-rot fungus *Postia placenta* (Hirosue et al. 2011; Ide et al. 2012; Ichinose 2013). A few individual P450s with interesting activities were also looked at more closely (Kasai et al. 2009; Syed et al. 2010; Kasai et al. 2010a; Kasai et al. 2010b; Chigu et al. 2010; Syed et al. 2011; Ichinose and Wariishi 2012; Syed et al. 2013b; Syed et al. 2013c; Hatakeyama et al. 2016; Sakai et al. 2018; Yang et al. 2018; Wang et al. 2019). However, the research about fungal P450s is still in its early stages and often shares ideas and limited information rather than comprehensive details on the P450 function. Generally, the biocatalytic repertoire of basidiomycetes as a whole remains greatly understudied (Schmidt-Dannert 2016). Compared to bacterial P450 enzymes, one reason for this might be the more challenging recombinant expression and scarce availability in general, as well as the lack of access to isolated enzymes. In part, this is due to the generally low expression of mostly membrane-bound enzymes and their need for several co-expressed interacting peptide chains to become enzymatically fully functional.

A comparison of the CYPomes of six model wood-degrading fungi revealed that 11 out of 68 P450 families were enriched, including CYP5035, CYP5136 and CYP5150 (Syed et al. 2014). While a few studies analysed enzymes of the latter two families (Syed et al. 2011; Ichinose and Wariishi 2012; Syed et al. 2013c; Hatakeyama et al. 2016), the function of CYP5035 is still inconclusive (Syed et al. 2014). Four members of its subfamilies A and B from *P*. *chrysosporium* were expressed in yeast and accepted naproxen, flavone or dehydroabietic acid to form yet unresolved products (Hirosue et al. 2011). However, no activity could be observed for the 13 other expressed CYP5035 enzymes of subfamilies A–E from the same fungus (Hirosue et al. 2011) or of subfamily F of *P*. *placenta* (Supplementary Table S1) (Ide et al. 2012). Hence, the knowledge about this enriched P450 family is largely insufficient and calls for further investigation.

In this study, putative P450 sequences from publicly available genomes of phylogenetically closely related (Krüger and Gargas 2004; Sotome et al. 2008; Seelan et al. 2015) white-rot fungi *Polyporus arcularius*, *Polyporus brumalis*, *Polyporus squamosus* and *Lentinus tigrinus* were extracted and their P450 families assigned. Thereby, it was noticed that the CYP5035 and CYP5136 families were enhanced even more than in any of the model white-rot fungi already analysed in the literature, except in *Ganoderma* species. By sketching their phylogeny in contrast to *P*. *chrysosporium* and other model basidiomycetes, the evolution of these enzyme families was studied in the systematic order of *Polyporales*. To fill part of the described functional gap, a representative group of nine CYP5035 sequences dispersed over the available CYP5035 subfamilies in *P*. *arcularius* were heterologously expressed in *Komagataella phaffii* (*Pichia pastoris*) and screened for activity towards a test set of structurally diverse substrates representing several different natural product classes. Thereby, a promiscuous CYP5035 was discovered.

## Materials and methods

All solvents and chemicals were purchased from Sigma-Aldrich/Merck (Steinheim/Darmstadt, Germany), VWR International (Fontenay-sous-Bois, France), Carl Roth GmbH (Karlsruhe, Germany) or Fisher Scientific (Loughborough, UK) in best available purity and were used as received without further purification. HPLC tubes were bought from Macherey-Nagel (Düren, Germany) and the corresponding caps and inserts from Bruckner Analysentechnik (Linz, Austria). An Agilent Technologies 1100 Series executed the HPLC analysis, and a Shimadzu GCMS-QP2010 SE instrument equipped with an AOC-20i/s autosampler and injector unit together with a Zebron ZB-5MSi capillary column (30 m × 0.25 mm × 0.25 μm, Phenomenex) performed the GC-MS measurements. OD values were determined with an Eppendorf BioPhotometer plus. The CYP5035 coding regions, identified from the publicly available databases, were ordered as double-stranded DNA fragments from TWIST Bioscience. Cells of *P. pastoris* with expressed and versatile P450 3A4 were obtained from bisy GmbH (Hofstaetten, Austria) and used as a positive control for biotransformations. These cells had been cultivated, then stored as frozen pellets at −80°C. Figures were generated in the programmes GraphPad Prism 8 and CS ChemDraw Ultra.

### Fungal CYPome determination

Publicly available protein sequences of the genomes of the following species were downloaded from the Joint Genome Institute (JGI) Genome Portal website (https://genome.jgi.doe.gov/portal/) of the US Department of Energy (Grigoriev et al. 2014):

- *Polyporus arcularius* (Varga et al. 2019) HHB13444 v1.0: Project: 1006899
  (https://mycocosm.jgi.doe.gov/Polar1/Polar1.home.html);
- *Polyporus brumalis* (Miyauchi et al. 2018) BRFM 1820 v1.0: Project: 1051563
  (https://mycocosm.jgi.doe.gov/Polbr1/Polbr1.home.html);
- *Polyporus squamosus* CCBS676 v1.0: Project 1108915
  (https://genome.jgi.doe.gov/portal/Polsqu1/Polsqu1.download.html);
- *Lentinus tigrinus* (Wu et al. 2018) ALCF2SS1-6 v1.0: Project 1020066
  (https://genome.jgi.doe.gov/portal/Lenti6_1/Lenti6_1.download.html).

In each case, the Files -> Annotation -> Filtered Models (‘best’) -> Proteins -> ‘*Species*’_GeneCatalog_proteins.aa.fasta.gz files were used.

Additionally, the necessary genome P450s sequences (Martinez et al. 2004; Martinez et al. 2009; Eastwood et al. 2011; Suzuki et al. 2012; Floudas et al. 2012; Morin et al. 2012; Binder et al. 2013) or CYPome statistics of the fungi *Phanerochaete chrysosporium* (Syed and Yadav 2012; Syed et al. 2014), *Phanerochaete carnosa* (Suzuki et al. 2012; Syed et al. 2014), *Agaricus bisporus* (Syed et al. 2014), *Postia placenta* (Ide et al. 2012; Syed et al. 2014), *Ganoderma* sp. (Syed et al. 2013a; Syed et al. 2014; Kües et al. 2015), *Serpula lacrymans* (Syed et al. 2014), *Trametes versicolor* (Syed and Mashele 2014), *Bjerkandera adusta* (Syed et al. 2013a), *Phlebia brevispora* (Syed et al. 2013a), *Heterobasidion irregulare* (Mgbeahuruike et al. 2017), *Phlebiopsis gigantea* (Hori et al. 2014), *Lignosus rhinoceritis* (Yap et al. 2014), *Ganoderma lucidum* (Chen et al. 2012; Kües et al. 2015) and *Ganoderma sinense* (Zhu et al. 2015) were obtained from the cited literature or accessed according to their instructions (Online Resource 1).

The CYPomes of *P*. *arcularius*, *P*. *brumalis*, *P*. *squamosus* and *L*. *tigrinus* (Online Resource 2) were determined according to the P450 identification and annotation strategy described by Syed and Mashele (2014) with a slight adjustment as the old BLAST server on the Cytochrome P450 Homepage (Nelson 2009) did not work and the new P450 BLAST page (http://www.p450.unizulu.ac.za/?page_id=21) had not been installed at the time:

1. Superfamily annotation of the protein sequences by the Batch CD-Search Tool of the National Center for Biotechnology Information (NCBI) (https://www.ncbi.nlm.nih.gov/Structure/bwrpsb/bwrpsb.cgi).
2. Verification of the P450 signature motifs ‘E-x-x-R’ and ‘C-x-G’ in the putative P450 sequences using the ScanProsite tool (https://prosite.expasy.org/scanprosite/).
3. P450 family assignment by applying the FCPB BLAST Search of the verified P450 protein sequence (http://p450.riceblast.snu.ac.kr/blast.php) (Moktali et al. 2012).
4. Verification of the P450 family assignment by alignment against already assigned P450 sequences of previous publications using the Protein BLAST option of the NCBI. P450 families were verified according to the 40% (family) and 55% (subfamily) sequence identity rules of the International P450 Nomenclature Committee (Nelson 2006).

Step 4 was of utter importance to assign less common P450 families correctly because the FCPB did not consider enzyme families of the *Ganoderma* macrofungus. P450 sequences that did not get a good match were left unassigned.

### Evolutionary P450 sequence analysis

Following the same strategy as Syed et al. (2014), evolutionary analyses of the desired P450 protein sequences were conducted in MEGA X (Kumar et al. 2018; Stecher et al. 2020). The evolutionary history was inferred using the minimum evolution method (Rzhetsky and Nei 1992). The evolutionary distances were computed using the Poisson correction method (Zuckerhandl and Pauling 1965) and are in the units of the number of amino acid substitutions per site. The minimum evolution tree was searched using the close-neighbour-interchange algorithm (Nei and Kumar 2000) at a search level of 1. The neighbour-joining algorithm (Saitou and Nei 1987) was used to generate the initial tree. All ambiguous positions were removed for each sequence pair (pairwise deletion option).

### CYP5035 enzyme expression in *Pichia pastoris*

Due to the advantages for the functional overexpression of membrane-bound eukaryotic P450 enzymes and success in co-expression experiments of foreign P450 genes with the P450 reductase of *P*. *pastoris* (PpCPR) described previously (Geier et al. 2012), representative *P*. *arcularius* P450 genes were expressed by *P*. *pastoris*. Nine CYP5035 sequences of *P*. *arcularius* were ordered as synthetic double-stranded DNA fragments from TWIST Bioscience, amplified and cloned into the expression vector (Supplementary Fig. S1) equipped with Zeocin resistance and a bidirectional promoter for co-expression of the PpCPR gene by Gibson assembly. The *P*. *pastoris* strain BSYBG11 (*aox1*Δ, MUT^S^), a derivative of *P*. *pastoris* strain BG08 of BioGrammatics Inc. (Carlsbad, USA) (Sturmberger et al. 2016), was transformed with the resulting linearised plasmids for genomic integration of the expression cassettes. Small-scale cultivations were done for the 7-methoxy-4-(trifluoromethyl)coumarin (MFC) demethylation assay and carried out following the deep-well plate (DWP) and induction protocols reported previously (Weis et al. 2004). For the substrate screening, cultivations were scaled up to 250-mL shake-flasks inoculating 45 mL BMD1 (pH 7.4), adding 5 mL BMM10 (pH 7.4) after 60 h (Weis et al. 2004) and three times further feeding with 0.5 mL methanol every 12 h. Having harvested and washed cells twice in 50 mM potassium phosphate buffer (pH 7.4), cells were resuspended in the same phosphate buffer until an OD_600_ of 100 was obtained. A cell broth volume of 400 μL was filled into each well of a DWP and 4 μL of the 100 mM compound stock solutions added to get a final substrate concentration of 1 mM. The biotransformations were carried out for 17 h at 28°C, 80% humidity and 320 rpm in a tilted orientation on the shaker to ensure maximal oxygen availability. After stopping the reaction with the addition of 300 μL of an acetonitrile/methanol (1:1; v/v) solution, the resulting mixture was vortexed and centrifuged and 200 μL of supernatant of each well were transferred into 96-well GreinerV plates for HPLC analysis. The biotransformations of all compounds were analysed by HPLC, except for squalene by GC-MS. Percentage conversions were calculated by peak area integration of new peaks that were not present in the negative control (Supplementary Fig. S2). Separation was carried out via a Kinetex C18 (100 Å; 50 × 4.6 mm; 2.6 μm) reverse-phase column. Water containing 0.1% acetic acid (A) and acetonitrile (B) was used for elution at 25°C in the following ratios: 0 min: A/B 80/20; 1 min: A/B 80/20; 1.01 min: A/B 50/50; 4 min: A/B 0/100; 5.50 min: A/B 0/100; 5.51 min: A/B 80/20; and 6.50 min: A/B 80/20. GC-MS analysis; equal volumes of dichloromethane containing 0.01% undecane were added to the biotransformations and after phase separation, the organic layer was dried with anhydrous Na_2_SO_4_. The following method (linear velocity of 39.5 cm s^−1^ using He carrier gas; total and column flow of 15.2 mL min^−1^ and 1.21 mL min^−1^, respectively; injection temperature of 250°C; split ratio of 9.1) was applied: 35°C for 5 min, 20°C min^−1^ to 300°C and 300°C for 5 min in a total run time of 23.25 min.

## Results

**Fig. 1:**
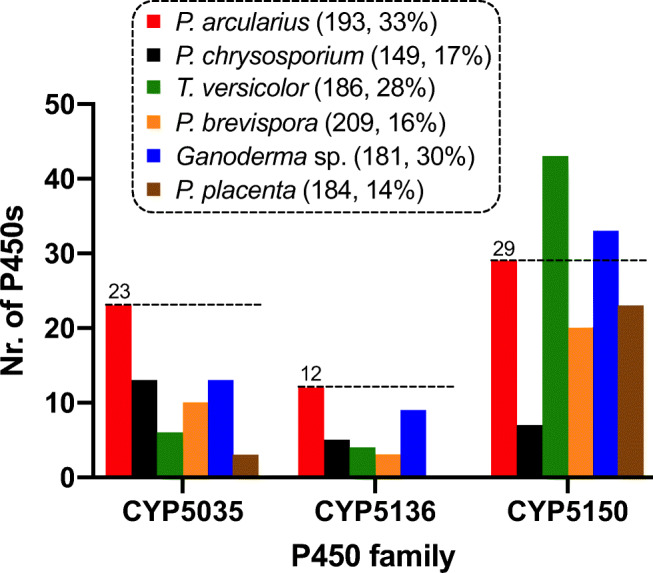
A comparison of the number of P450 genes of the families CYP5035, 5136 and 5150 identified in the genome of *P*. *arcularius* (Parc) with several model white- (*P*. *chrysosporium*, *T. versicolor*, *Phlebia brevispora*, *Ganoderma* sp.) and brown-rot fungi (*P*. *placenta*, *Ppla*) is shown. The total number of P450s and the percentage of these three families in comparison to all P450s in the translated genome of each species are given in parenthesis. A more detailed CYPome comparison is provided in Online Resource 1

Using the publicly available genome and translated protein sequences from the JGI Genome Portal website (Grigoriev et al. 2014), a genome-wide search for putative P450s in *P*. *arcularius* (Supplementary Fig. S3) was carried out following the identification and annotation strategy of Syed and Mashele (2014) (Online Resource 2) with a few adjustments as outlined in the ‘Materials and methods’ section. The CYPome of *P*. *arcularius* showed a similar collection of P450s to that of *Ganoderma* sp., primarily owing to the presence of numerous CYP5359 and a few CYP5144 (Online Resource 1). Likewise, a CYPome comparison between *P*. *arcularius* and well-known model white- (e.g. *P*. *chrysosporium*) and brown-rot (e.g. *P*. *placenta*) fungi analysed extensively by previous studies (Syed et al. 2013a; Syed et al. 2014; Syed and Mashele 2014) revealed an unusually large number of CYP5035 (23) and CYP5136 (12) in this fungus: approximately 2-fold more (13; 5) than in *P*. *chrysosporium* (Fig. 1). In fact, even when extending the comparison to a total of 17 wood-degrading fungi, *P*. *arcularius* had the highest percentages of these two P450 families in its CYPome (Supplementary Figs. S4 and S5). Only the absolute number of CYP5035 and CYP5136 was surpassed slightly by *Ganoderma sinense* and *P*. *brumalis*, respectively (Supplementary Figs. S6 and S7) (Zhu et al. 2015). Additionally, CYP5150 coding gene sequences were frequent in the genome (Supplementary Fig. S8), with the percentage of this family in the genome higher in *Ganoderma* species and *Trametes versicolor* (Supplementary Fig. S9).

**Fig. 2:**
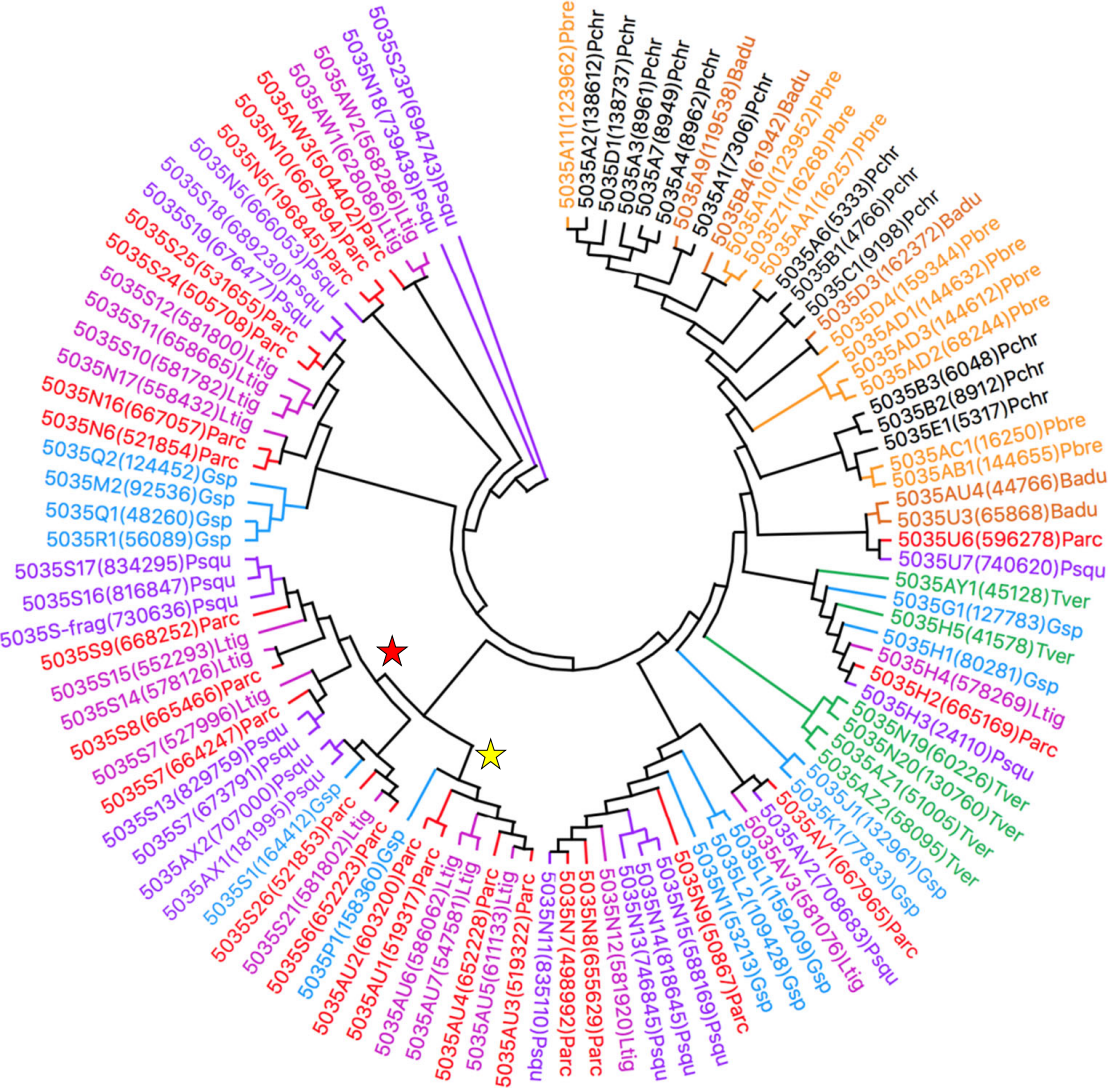
A minimum evolution tree of the CYP5035 family involving 103 amino acid sequences from eight different organisms. The phylogeny of CYP5035 enzymes of the fungus *P*. *arcularius* (Parc; red) compared to related species *L*. *tigrinus* (Ltig; violet) and *P*. *squamosus* (Psqu; purple) and the other model white-rot fungi *Ganoderma* sp. (Gsp; blue), *T*. *versicolor* (Tver; green), *P*. *chrysosporium* (Pchr; black), *B*. *adusta* (Badu; dark orange) and *P*. *brevispora* (Pbre; orange) in order to get an insight into the evolution of this P450 family. The yellow or red stars indicate a diversification process and new branch of the P450 families compared to *Ganoderma* sp., respectively. The tree was constructed using the close-neighbour-interchange algorithm in MEGA X. An extended tree can be found in Supplementary Fig. S15

**Fig. 3:**
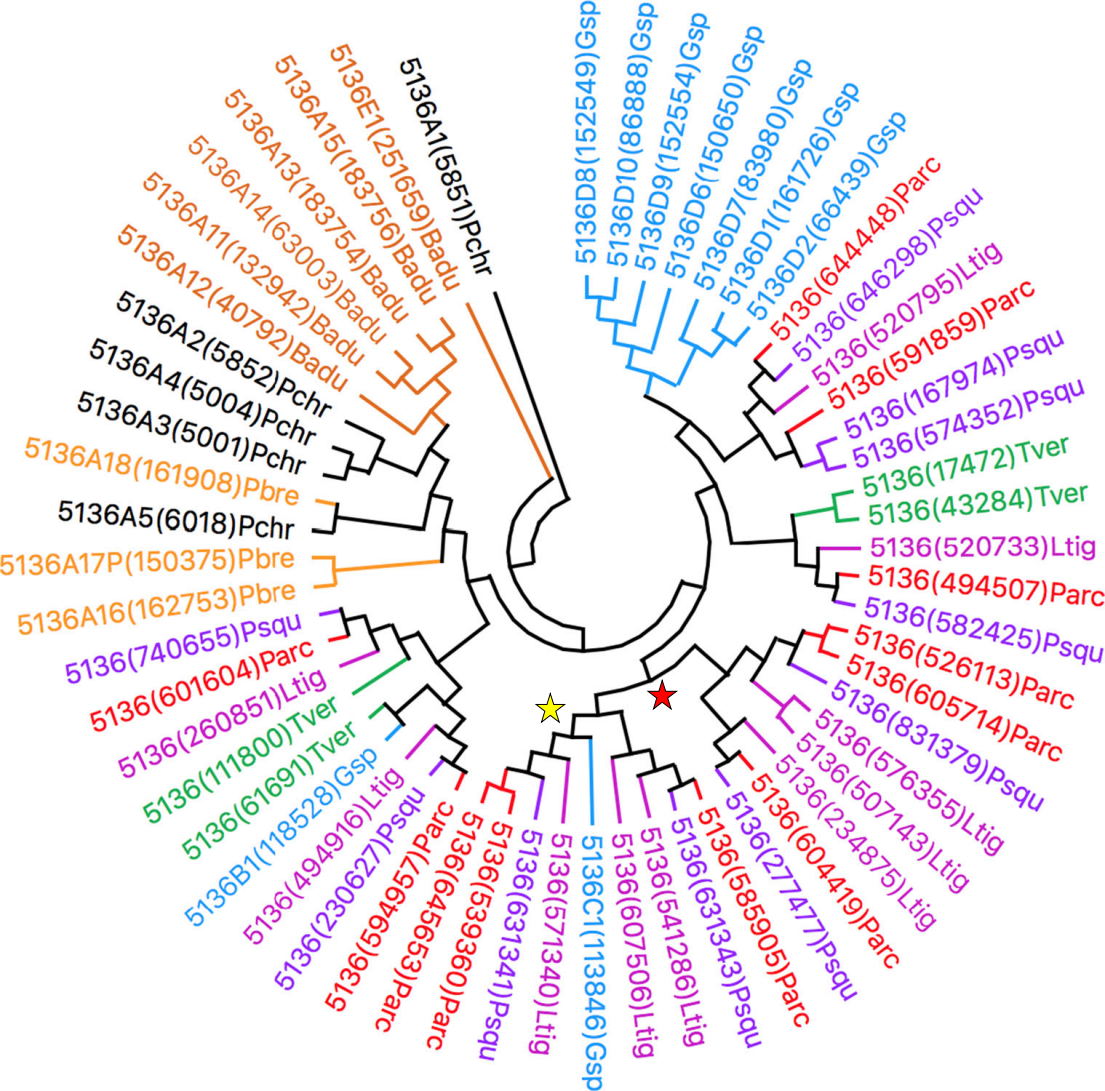
Displayed is a minimum evolution tree of the CYP5136 family involving 58 amino acid sequences from eight different organisms. Phylogeny of CYP5136 enzymes of fungus *P*. *arcularius* (Parc; red) compared to related species *L*. *tigrinus* (Ltig; violet) and *P*. *squamosus* (Psqu; purple) and the other model white-rot fungi *Ganoderma* sp. (Gsp; blue), *T*. *versicolor* (Tver; green), *P*. *chrysosporium* (Pchr; black), *B*. *adusta* (Badu; dark orange) and *P*. *brevispora* (Pbre; orange) in order to get an insight into the evolution of this P450 family. The yellow or red stars indicate a diversification process and new branch of the P450 families compared to *Ganoderma* sp., respectively. The tree was constructed using the close-neighbour-interchange algorithm in MEGA X. An extended tree can be found in Supplementary Fig. S16

When consulting literature articles for the relative phylogeny of *P*. *arcularius* to that of white-rot fungal species for which CYPomes had been analysed previously (Justo and Hibbett 2011; Floudas et al. 2012; Binder et al. 2013), the *Ganoderma* macrofungus indeed turned out to be the fungus most closely related (Supplementary Fig. S10). Perhaps the evolved enrichment of CYP5035 and CYP5136 had its origin at approximately this branching point in fungal diversification and continued downstream to *P*. *arcularius*. In order to answer this hypothesis, the CYPomes of *P*. *brumalis* (Supplementary Fig. S11) (Miyauchi et al. 2018), *P*. *squamosus* (Supplementary Fig. S12) and *L*. *tigrinus* (Supplementary Fig. S13) (Wu et al. 2018) were determined according to the aforementioned genome-wide P450 identification strategy. The selected fungal genomes unveiled a similarly high number of CYP5035 and CYP5136 (Online Resource 1). The computation of minimum evolution trees of both P450 families shown in Figs. 2 and 3, respectively, further supported the proposed evolutionary theory. Due to its close relationship to *P*. *arcularius* (Supplementary Fig. S14), *P*. *brumalis* was only included together with further white- and brown-rot fungi in extended phylogenetic trees (Supplementary Figs. S15 and S16), which contributed towards the same conclusions.

The presence of the large number of CYP5035 sequences in the genome of *P*. *arcularius* awoke our interest in studying this P450 family in more detail. It was thus decided to pick a small, representative selection of nine CYP5035 sequences of *P*. *arcularius* distributed among the available subfamilies (Supplementary Table S1, Online Resource 2) to express them in *P*. *pastoris* and to test their activities and substrate scope employing recombinant whole-cell biotransformation. These nine CYP5035 were cloned into bidirectional co-expression plasmids (Vogl et al. 2018) together with *P*. *pastoris*’ native P450 reductase (Supplementary Figure S1). Fourteen transformants of each CYP5035 variant were screened for activity using the MFC demethylation assay (Donato et al. 2004) to select the best clonal variants. Although only CYP5035S7 was active when employing the simple MFC screening assay, the formation of a blue colour upon conversion of indole to indigo (Çelik et al. 2005) by both CYP5035S7 and CYP5035H2 indicated successful expression and oxidative activity also for other expressed P450 genes, which encouraged us to move forward with randomly selected individual transformants for each of the enzymes other than CYP5035S7 and test alternative substrates by chromatographic analysis. For this substrate specificity screening, the selected transformants were cultivated and applied in whole-cell biotransformation experiments with over 40 structurally diverse and complementary compounds of eight different natural product classes (terpenes, steroids, alkaloids, stilbenoid and flavonoid backbones, phenylpropanoids, fatty acid derivative and coumarins) and also pharmaceuticals, (nitrogen-containing) polycyclic aromatic hydrocarbons ((N)PAHs) and other chemicals. Due to its known broad substrate acceptance, the human P450 3A4 co-expressed with its human CPR in *P*. *pastoris* was used as a positive control for all biotransformations (Fessner et al. 2020). As a negative control, an empty vector strain only overexpressing the yeast’s intrinsic P450 reductase was used to identify a possible involvement of intrinsic *P*. *pastoris* enzymes in the substrate conversions.

**Fig. 4:**
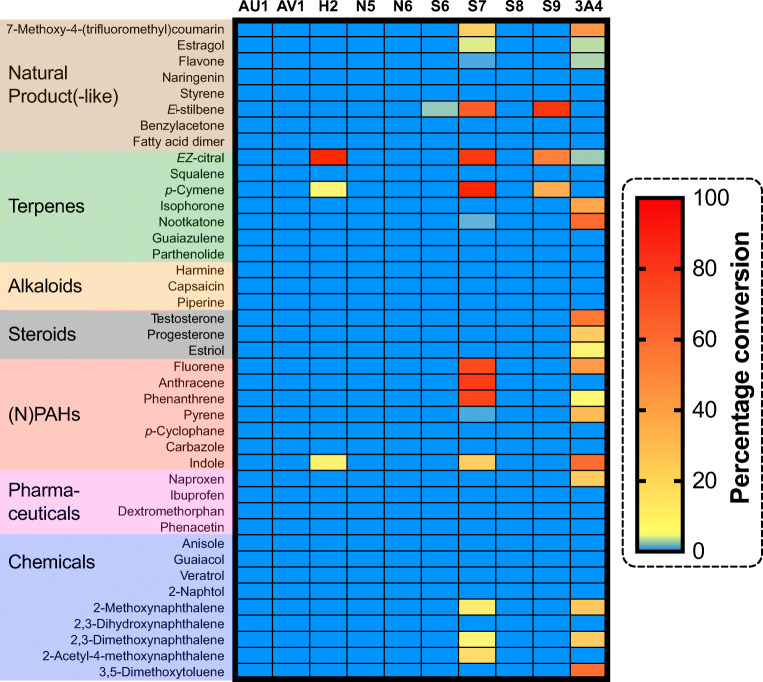
This heat map illustrates the result of the recombinant whole-cell activity screening of *P*. *pastoris* transformants expressing nine different CYP5035 enzymes of *P*. *arcularius* and the positive control co-expressing human P450 3A4 together with human CPR. An empty vector strain overexpressing the yeast’s intrinsic P450 reductase only was used as a negative control to identify possible background conversions caused by intrinsic *P*. *pastoris* enzymes. The percentage conversions of the initial 1 mM substrate concentrations were calculated from peak integration of newly appeared peaks in HPLC profiles (Supplementary Fig. S2). Only the biotransformation of squalene was analysed by GC-MS. Reaction conditions were as follows: OD600 of 100, 28°C, 320 rpm and 1 mM substrate concentration

**Fig. 5:**
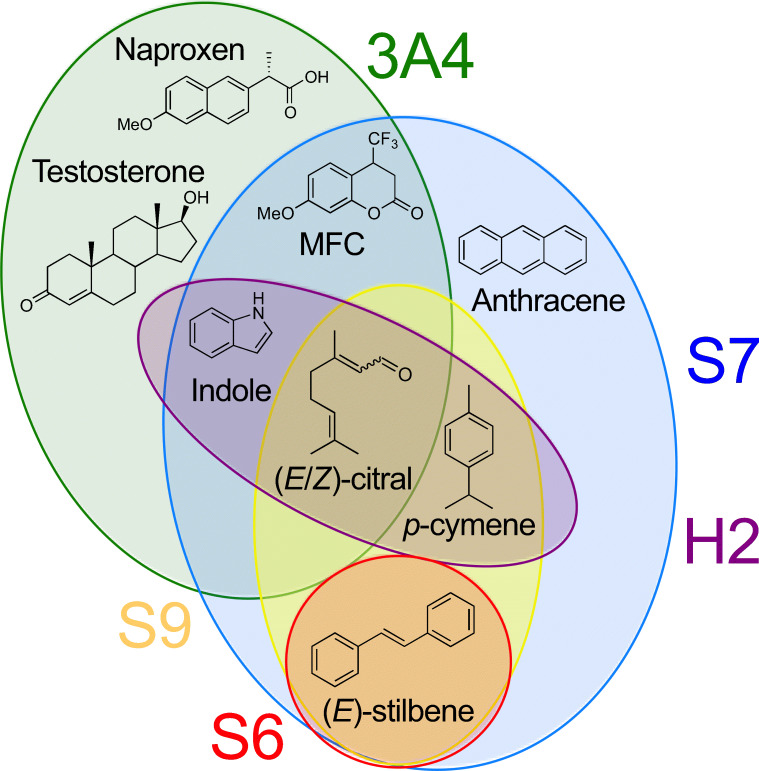
Venn diagram illustrating the catalytic promiscuity of several accepted substrates of the CYP5035 candidates and human P450 3A4 used for the whole-cell activity screening displayed in Fig. 4. Only a fraction of the substrates tolerated by only one or both of CYP5035S7 and P450 3A4 is shown

The heat map in Fig. 4 illustrates the activity pattern that was obtained from the HPLC or GC analysis. Notably, the observed activities imply different substrate scopes even among P450s of the same subfamily CYP5035S. While the three enzymes CYP5035H2, -S6 and -S9 only converted at least one of (*E*)-stilbene, (E/Z)-citral, *p*-cymene and indole, the fourth variant CYP5035S7 demonstrated a much larger substrate scope being active especially on PAHs and terpenes but also across other natural product classes such as the phenylpropanoid estragol and the stilbenoid-backbone (*E*)-stilbene (Fig. 5). In contrast, cells with expression constructs for CYP5035S8 and four other individual CYP5035 genes did not show any activity whatsoever, and their expression constructs were not further studied.

Having identified the promiscuity of CYP5035S7, a BLAST search for hits with this enzyme sequence against the personal P450 collection of David Nelson encompassing 102,000 sequences from JGI was performed to locate orthologous CYP5035 or similar sequences in other fungi (Online Resource 3). A total of 314 sequences in 36 different fungal species were longer than 450 amino acids and aligned with >40% identity, indicating an allocation to the same CYP5035 family, though only sequences of fungi used in this study were found to belong to the same subfamily with >55% identity. Hence, better allocation was achieved by blasting the 314 sequences each against all CYP5035 sequences named so far (Online Resource 3). The results were sorted by subfamily, percentage identity and species.

## Discussion

*P*. *arcularius* was selected for this work because in 2019, a study took the fungus into closer consideration as one of the candidates with high potential for organic pollutant degradation (Dao et al. 2019), and in the same year, its genome was sequenced (Varga et al. 2019). The observed enrichment of CYP5035, CYP5136 and CYP5150 in the investigated white-rot *Polyporales* pointed towards a higher diversity of P450s compared to other white-rot fungi. Most likely, such enrichments happened in order to adapt to harsher conditions in new ecological niches and to detoxify the high diversity of degradation products in such a world of different available carbon sources. The increase in functional P450s of these two families must have secured some evolutionary advantage (Syed et al. 2014; Kües et al. 2015). Former studies had clearly classified CYP5136 and CYP5150 as enzymes participating in the fungal defence mechanism and metabolic diversity degrading both plant material and xenobiotics. Equipped with broad substrate scopes, members of these P450 families were able to oxidise hydrocarbons, plant chemicals, steroids and pharmaceuticals (Kasai et al. 2010a; Syed et al. 2011; Ichinose and Wariishi 2012; Syed et al. 2013c; Syed et al. 2014; Hatakeyama et al. 2016; Lu et al. 2019). However, more limited activity information was available for CYP5035 (Supplementary Table S1). The fact that nonwood-degrading basidiomycetes conversely only possessed a few P450 genes, and these three enriched P450 families were completely absent in their genomes, strengthened their results (Syed et al. 2014). On the other hand, the heterologous expression of CYP5150L8 from *G*. *lucidum* was recently found to enable the biosynthesis of a ganoderic acid in *S*. *cerevisiae* (Wang et al. 2018). Therefore, the observed diversity of P450s in these studied organisms also suggests the essential roles of those enzymes in the synthesis of natural products.

This study aimed at investigating the functional potential of the enriched CYP5035 genes that might harbour an evolutionary advantage for some fungal species, keeping in mind the possibility of successful bioprospecting for innovative P450 enzymes useful for synthetic and industrial purposes. For efficient heterologous expression, the yeast *P*. *pastoris* was used as the host organism due to previous success with similar enzymes (Syed et al. 2010; Syed et al. 2011; Geier et al. 2012; Syed et al. 2013c) and a broad toolbox for complex protein expressions such as stepwise inducible strong promoters and expression strategies established in our laboratory (Vogl and Glieder 2013; Vogl et al. 2014; Weninger et al. 2015; Vogl et al. 2016; Vogl et al. 2018) and the advantage of the presence of an intrinsic, fungal P450 reductase (CPR) at a low abundance of intracellular P450s. Overexpression of intrinsic yeast CPR demonstrated beneficial effects for heterologous eukaryotic P450 expression before (Braun et al. 2012). Based on literature results undertaking CYPome functionality studies, also the native CPR of the yeast *Saccharomyces cerevisiae* generally seemed to couple well with basidiomycete P450s across different families (Kasai et al. 2009; Kasai et al. 2010a; Kasai et al. 2010b; Hirosue et al. 2011; Nazir et al. 2011; Ide et al. 2012). It was thus presumed that the same would apply to the related yeast *Pichia pastoris*, which had proven to be an excellent host for P450 expression with the potential for upscaling in a bioreactor (Martinez and Rupashinghe 2013; Byrne 2015; Fessner et al. 2020). Indeed, the functional screening confirmed the expected functional interaction with the co-expressed PpCPR.

Only a small selection of nine representative CYP5035 monooxygenases of *P*. *arcularius* was picked in order to balance efforts to cover the high enzymatic diversity representatively on the one hand and the screening efforts faced for the many substrates per enzyme planned on the other. In the end, this limited CYP5035 selection was sufficient to give a representative idea of the enzyme family’s capabilities, which revealed a much broader substrate acceptance than anticipated so far. Especially, CYP5035S7 showed a broad substrate scope encompassing PAHs and several natural product classes, offering itself as an attractive P450 for natural product modification. The high conversion of PAHs (82–87%; Fig 4) is in line with several articles that identified white-rot fungal P450s of different families with remarkable PAH conversion abilities towards various ring sizes (Syed et al. 2010; Syed et al. 2011; Syed et al. 2013b). Particularly, striking was the mutual conversion of *p*-cymene, (*E*)-stilbene and (*E*/*Z*)-citral by three different CYP5035 enzymes each (Fig. 5) because carvacrol and resveratrol, which are derivatives of the former two compounds, and (*E*/*Z*)-citral itself are known fungicidal agents (Yoneyama and Natsume 2010; Jian et al. 2016).

Since only CYP5035S7 was active in the fluorescence assay employing substrate MFC for activity screening, transformants of the other tested CYP5035 expression strains were picked randomly encouraged by the visible blue colour upon indigo formation also by CYP5035H2, which suggested possible activity of the enzymes beyond the MFC demethylation assay. Especially for the highly selective families of P450 enzymes, surrogate screening substrates only provide insufficient information about the real biocatalytic potential. This subset of nine new and different P450 genes was expected to provide also some information about which fraction of new fungal P450 genes can be expected to be functionally expressed in such blind discovery approach. Previously published studies demonstrated that functional expression of basidiomycete P450s is challenging and commonly based on trial and error (Schmidt-Dannert 2016). For five of the nine individual CYP5035 genes, which did not show any activity, it remains unclear if the lack of activity was due to the lack of a suitable substrate in the employed test set or if functional overexpression using the *P*. *pastoris*/PpCPR system failed. Due to the usually low expression of recombinant membrane-bound P450s, the lack of suitable antibodies and the limited information about P450 functionality, which can be obtained from P450 quantification by carbon monoxide spectroscopy in the case of multicomponent membrane-bound P450 systems, those expression constructs were not further studied in this functional approach for P450 screening. Ultimately, authentic activity information and the efficient bioprospecting for fungal enzymes towards industrial application rely on efficient heterologous expression (Mitrovic and Glieder 2015).

Interestingly, none of the active enzymes tested here accepted any of the tested active pharmaceutical ingredients (APIs), although three of four active CYP5035 of *P*. *chrysosporium* converted naproxen in a previous study (Hirosue et al. 2011). These observations highlight the deviating nature of P450s of phylogenetically similar species or even the same subfamily with sequence identities of >60% but different chemoselectivity. However, this study identified with CYP5035S7, an interesting new P450 that showed in this study a similarly versatile substrate acceptance as the broadly active human P450 3A4, which initiates detoxification pathways for many compounds in the human liver. The high conversion rates (82–100% for red squares; Fig. 4) for the test substrates in the applied substrate screening procedure suggest sufficient expression and activity for preparative applications just as demonstrated for human P450 3A4 whole-cell biocatalyst before (Fessner et al. 2020). At the same time, different candidates of CYP5035 variants have now been shown to be multifunctional with a diverse catalytic activity oxidising PAHs, pharmaceuticals and various plant materials encompassing fungicidal agents, which strongly suggests that CYP5035 are part of the fungal detoxification mechanism just like CYP5136 and CYP5150.

**Fig. 6:**
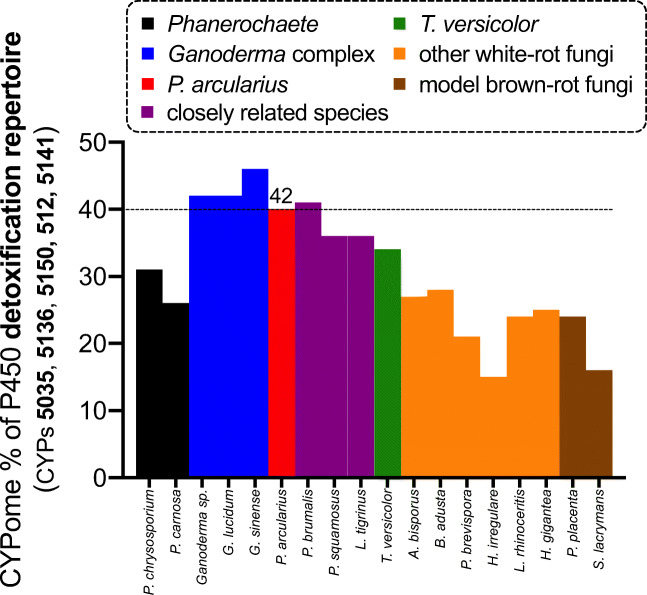
A comparison of the number of P450s considered to be part of the detoxification repertoire (CYP5035, CYP5136, CYP5150, CYP512 and CYP5141) of various white-rot fungi as a percentage of the total number of P450s in the fungus’s genome. The white-rot fungi focused on by this study possess an unusually high number of such detoxifying P450s compared to model white- and brown-rot fungi

In addition to the other two enriched families CYP5136 and CYP5150 mentioned earlier, this further increases the percentage of those enriched P450s with a detoxifying function to a third of the CYPome in the genome of *P*. *arcularius* (Supplementary Fig. S17). Apparently, *P*. *arcularius* and other species following the phylogenetic ladder up to the *Ganoderma* complex have an extensive repertoire of such xenobiotic-biodegrading P450s. As shown in Fig. 6, this phenomenon is preserved even when including P450 families CYP512 and CYP5141, which have also been flagged with xenobiotic-degrading functions (Syed et al. 2014). Interestingly, *T*. *versicolor* as the phylogenetic parent of the *Ganoderma* macrofungus also possesses an enhanced collection of such P450s in its genome compared to the other white-rot fungi. However, this is mainly due to the unmatched number of CYP5150 (Fig. 1, Supplementary Figs. S8 and S9).

Computing the phylogeny of both P450 families with minimum evolution trees made it possible to follow the evolutionary tree of this enrichment up to the *Ganoderma* complex, where the diversification of CYP5035 members might have branched off. The CYP5136 family expansion was found to have occurred separately for *Ganoderma* species and was intensified later. These conclusions were drawn due to the following observations: (1) the closer phylogenetic P450 relation within the group of *P*. *arcularius*, *P*. *squamosus* and *L*. *tigrinus* to *Ganoderma* sp. than to white-rot fungi *P*. *chrysosporium*, *P*. *brevispora*, *T*. *versicolor* or *Bjerkandera adusta* became clearly visible. (2) An intensified diversification process of the P450 families can be noticed especially in Fig. 2 for CYP5035 in parallel to almost all of *Ganoderma* sp. enzymes, often yielding higher numbers of homologous P450s (examples marked by yellow stars). (3) Additionally, from some common nodes, new branches diverged, which do not contain an enzyme member of *Ganoderma* sp. (examples marked by red stars). (4) Therefore, the CYP5035 family expansion likely happened before speciation with *Ganoderma* as the starting point and continued further downstream. (5) In contrast, diversification of CYP5136 occurred separately in the case of *Ganoderma* as indicated by the lack of ortholog pairs and started only later in time.

It remains unclear whether the *Ganoderma* macrofungus itself really is the starting point or merely one of the species in the row of the evolution of CYP5035 enrichment. However, the range of 13–26 members of CYP5035 and 7–9 members of CYP5136 within *Ganoderma* sp., *G*. *sinense* and *Ganoderma lucidum* is one argument for the former. The starting point of the diversification of CYP5136 is also unknown. Syed et al. suggested P450 gene duplication due to environmental adaptation as the origin for such family expansions (Syed et al. 2014). For example, the ortholog P450 of CYP5035C1 of *P*. *chrysosporium* was duplicated several times in *P*. *carnosa*, indicating some evolutionary advantage.

The sequence alignment search of CYP5035S7 against 102,000 P450 sequences from JGI only revealed orthologous enzymes (>80% ID) within the fungal species used in this study and generally indicated relatively small amounts of CYP5035 in each of the >30 other fungal species (Online Resource 3). Only *Dichomitus squalens* and *Earliella scabrosa* possessed a considerable number of CYP5035 sequences, suggesting that large numbers of this P450 family are rather rare. In addition, 38 of the 314 blasted sequences had <55% identity and will belong to yet undefined CYP5035 subfamilies.

In combination with the observed reasonable success rate for functional recombinant expression of those membrane-bound multicomponent P450 complexes and the phylogenetic analysis of CYP5035 and CYP5136, this BLAST result provides a valuable starting point for future bioprospecting for xenobiotic-degrading P450s with activity towards plant compounds similar or complementary to *P*. *arcularius*. The large repertoire of such detoxification monooxygenases within the white-rot fungal genomes shown in Fig. 6 incites further interest because it may provide a versatile toolbox of white-rot fungal P450 enzymes for natural product modification. Information about the activities of only three subfamilies were uncovered to date, although already >50 different CYP5035 subfamilies were categorised and will increase further. Hence, despite the efforts of this study, there remains a large functional gap and an unhidden catalytic potential for CYP5035.

Despite their biosynthetic potential (Fessner 2019), the majority of P450s remains the so-called functionally uncharacterised ‘orphan P450s’ (Kelly and Kelly 2013) due to the shortage of studies investigating them (Durairaj et al. 2016) and the expression difficulties (Schmidt-Dannert 2016) also observed in this study and the sheer amount of sequences available in the sequenced genomes (Ferrer et al. 2016). Therefore, this study aimed at and substantially helped to obtain more information about the function of the hardly studied CYP5035. A surprising multifunctional enzyme CYP5035S7 from *P*. *arcularius* was identified holding a versatile synthetic potential that remains to be investigated further in lab- and pilot-scale preparative application experiments.

## Supplementary Information

ESM 1 (PDF 5011 kb)

ESM 2 (XLSX 44 kb)

ESM 3 (XLSX 172 kb)

ESM 4 (XLSX 148 kb)

## Data Availability

The data supporting the findings of this study are available within this article and the supplementary materials. The fungal genome data, on which this study is based, are publicly available online as outlined in the ‘Materials and methods’ section.

## Code availability

Any software used in this study for genomic data processing is publicly available online via the links provided in the ‘Materials and methods’ section.
