# Supplementary material for: Evolution and enrichment of CYP5035 in Polyporales: functionality of an understudied P450 family
Source: Appl Microbiol Biotechnol. 2021 Aug 30;105(18):6779–92. doi: 10.1007/s00253-021-11444-2 (PMC8426240; doi:10.1007/s00253-021-11444-2)
Supplement: Supplementary file 1 — (PDF 5011 kb) [file 253_2021_11444_MOESM1_ESM.pdf]

Applied Microbiology and Biotechnology

Supporting Information

for

Evolution and Enrichment of CYP5035 and CYP5136 in *Polyporales*: Functionality of an understudied P450 family

Nico D. Fessner,[1] David R. Nelson[2] and Anton Glieder[1,*]

1 Institute of Molecular Biotechnology, Graz University of Technology, NAWI Graz, Petersgasse 14/3, Austria
2 Department of Microbiology, Immunology and Biochemistry, University of Tennessee Health Science Center, Memphis, TN 38163, USA

* corresponding author: Anton Glieder
E-mail: a.glieder@tugraz.at
phone: (+43)-316-873-4074
Fax: (+43)-316-873-9302

Table S1: Displayed are all CYP5035 expressed here and in prior literature so far together with their tested substrate tolerance, host fungus and corresponding reference.

Reference	Fungus	**CYP5035…**	**Sequence ID**	**Substrate**
10.1016/j.bbrc.2011.02.121	*Phanerochaete chrysosporium*	A1	7306	naproxen
		A2	138612	naproxen
		A3	8961	?
		A4	8962	?
		A5	138737	flavone
		A6	5333	?
		A7	8949	?
		B1	–	?
		B2	8912	naproxen & abietic acid
		B3	6048	?
		C1	9198	?
		D1	–	?
		E1	5317	?
10.1007/s00203-011-0753-2	*Postia placenta*	F1	112190	?
		F2	129155	?
		F3v1	89499	?
		F3v2	89499	?
This study	*Polyporus arcularius*	H2	665169	(*EZ*)-citral, *p*-cymene, indole
		N5	196845	?
		N6	521854	?
		S6	652223	(*E*)-stilbene
		S7	664247	**multi-functional**
		S8	665466	?
		S9	668252	(*E*)-stilbene, (*EZ*)-citral, *p*-cymene
		AU1	519317	?
		AV1	667965	?

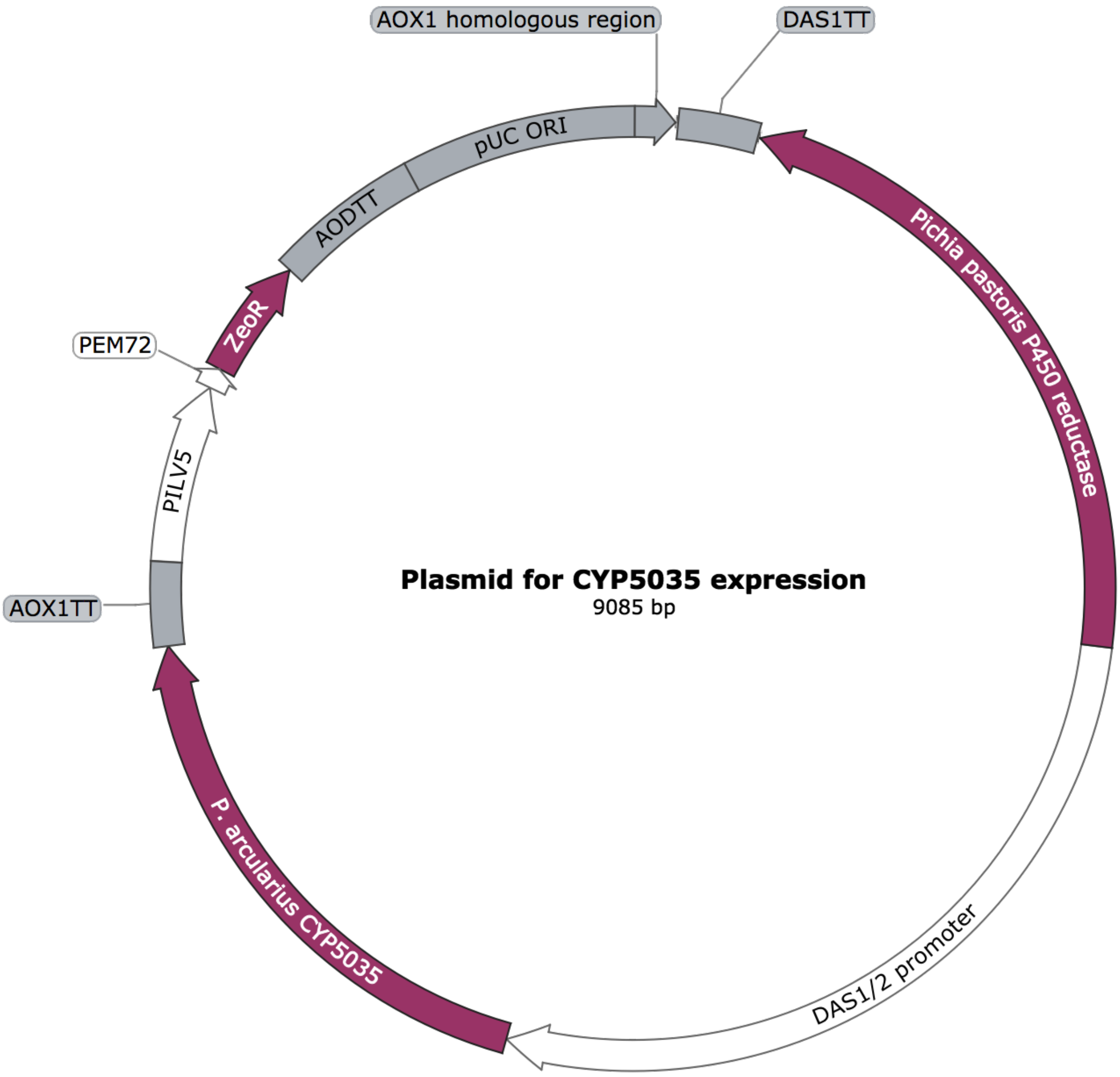

Fig. S1: Plasmid used for the coexpression of the CYP5035 enzymes and *P. pastoris*' native P450 reductase enzyme in the yeast.

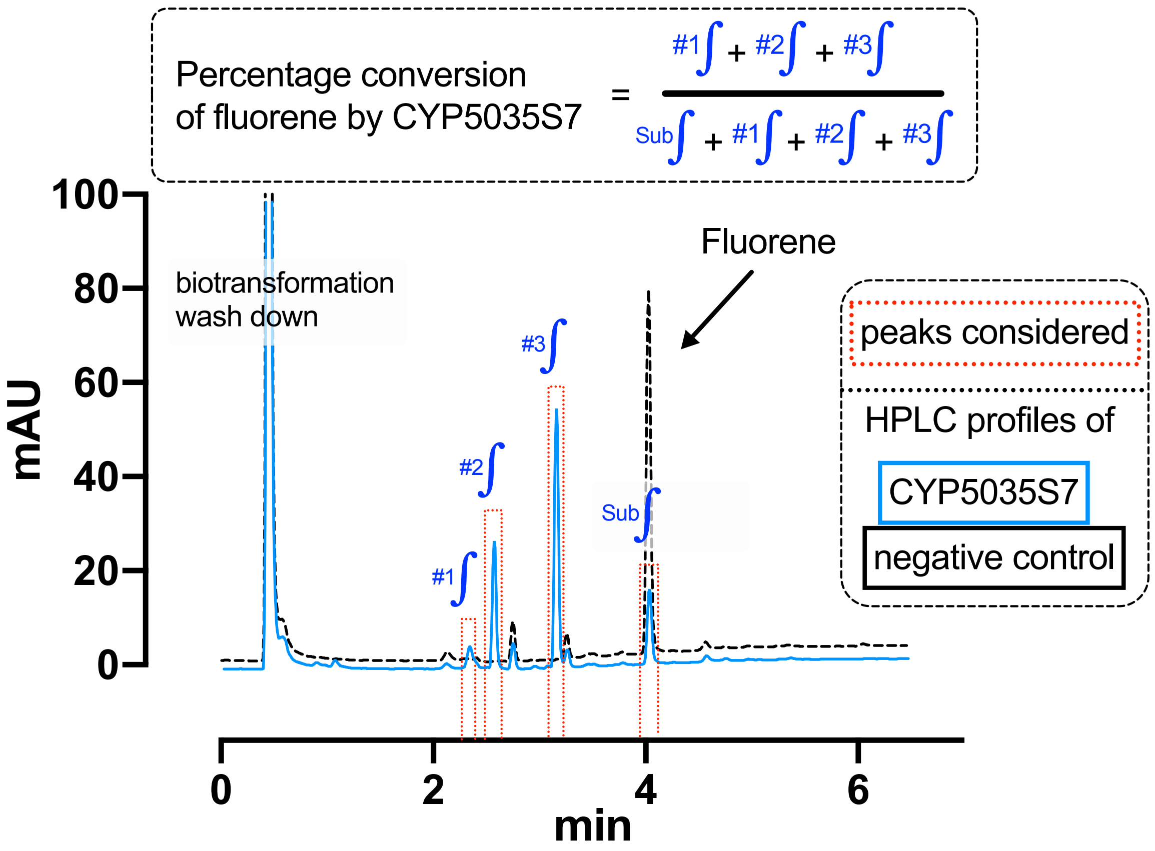

Fig. S2: Displayed are the HPLC biotransformation profiles of CYP5035S7 and the empty vector control to show the general procedure of how the percentage conversions of each compound listed in Fig. 4 were calculated. Peaks were integrated and the fraction of the combined areas of new peaks that were not present in the negative control divided by the former plus the substrate peak area.

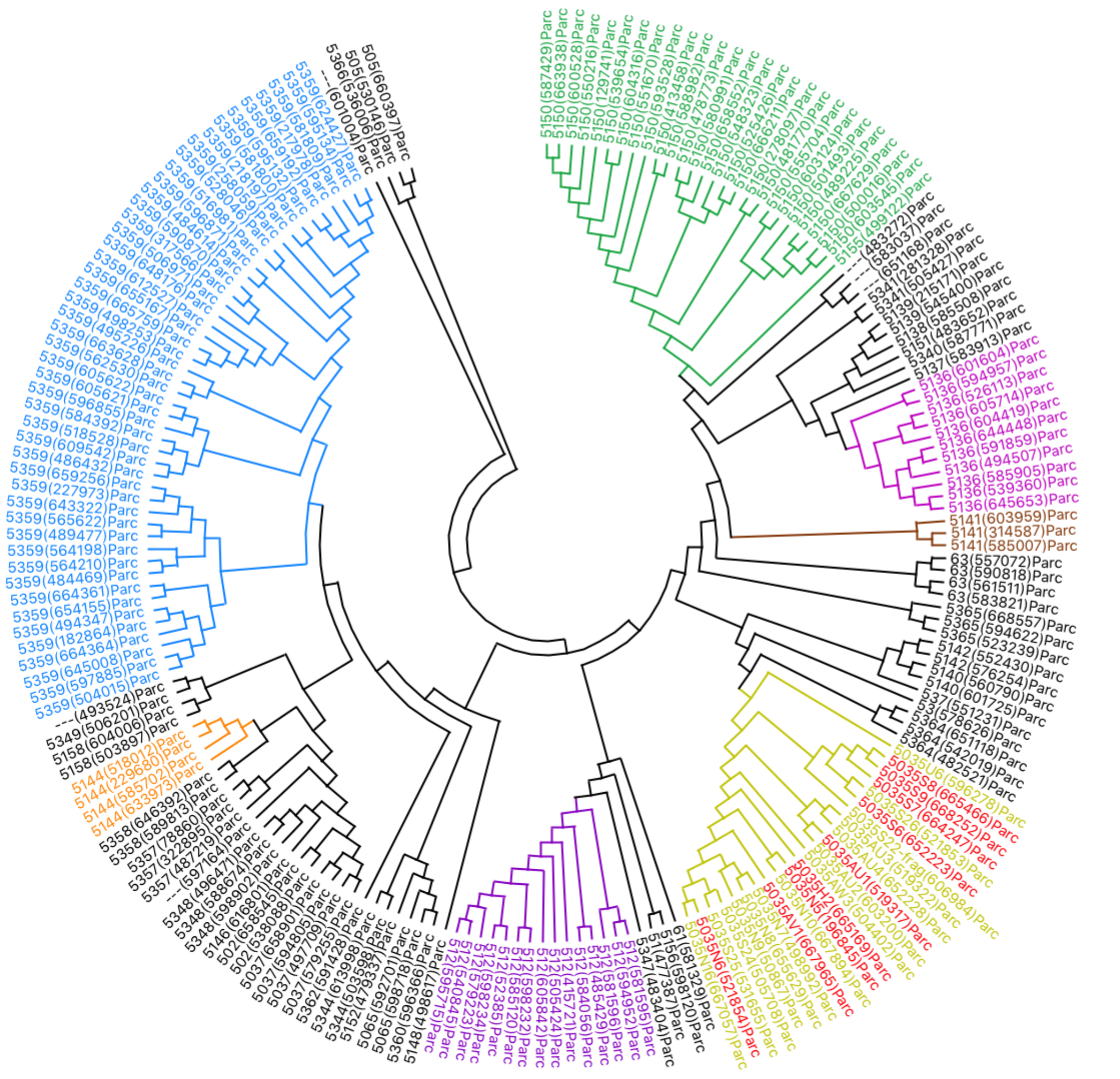

Fig. S3: A minimum evolution tree of the P450ome of *P. arcularius* involving 193 amino acid sequences. CYP512 (purple), CYP5035 (dark yellow), CYP5136 (violet), CYP5141 (brown), CYP5144 (orange), CYP5150 (green) and CYP5359 (blue) have been coloured. The P450 nomenclature of the CYP5035 selected and expressed for a functional screening is shown in red. The tree was constructed using the close-neighbour-interchange algorithm in MEGA X.

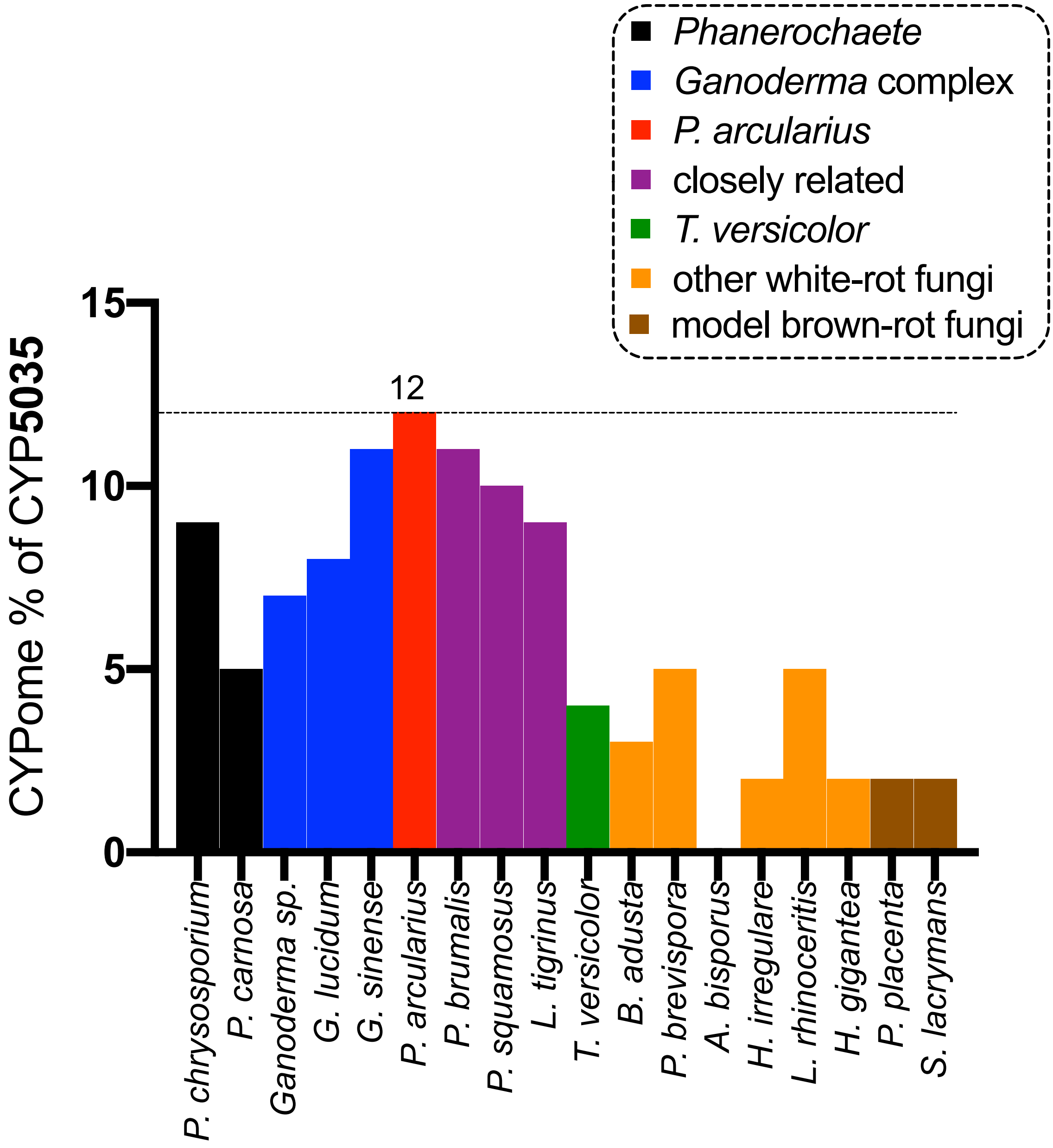

Fig. S4: Comparison of the number of CYP5035s as a percentage of the total number P450s in the genome of *P. arcularius* versus a variety of white- and brown-rot fungi.

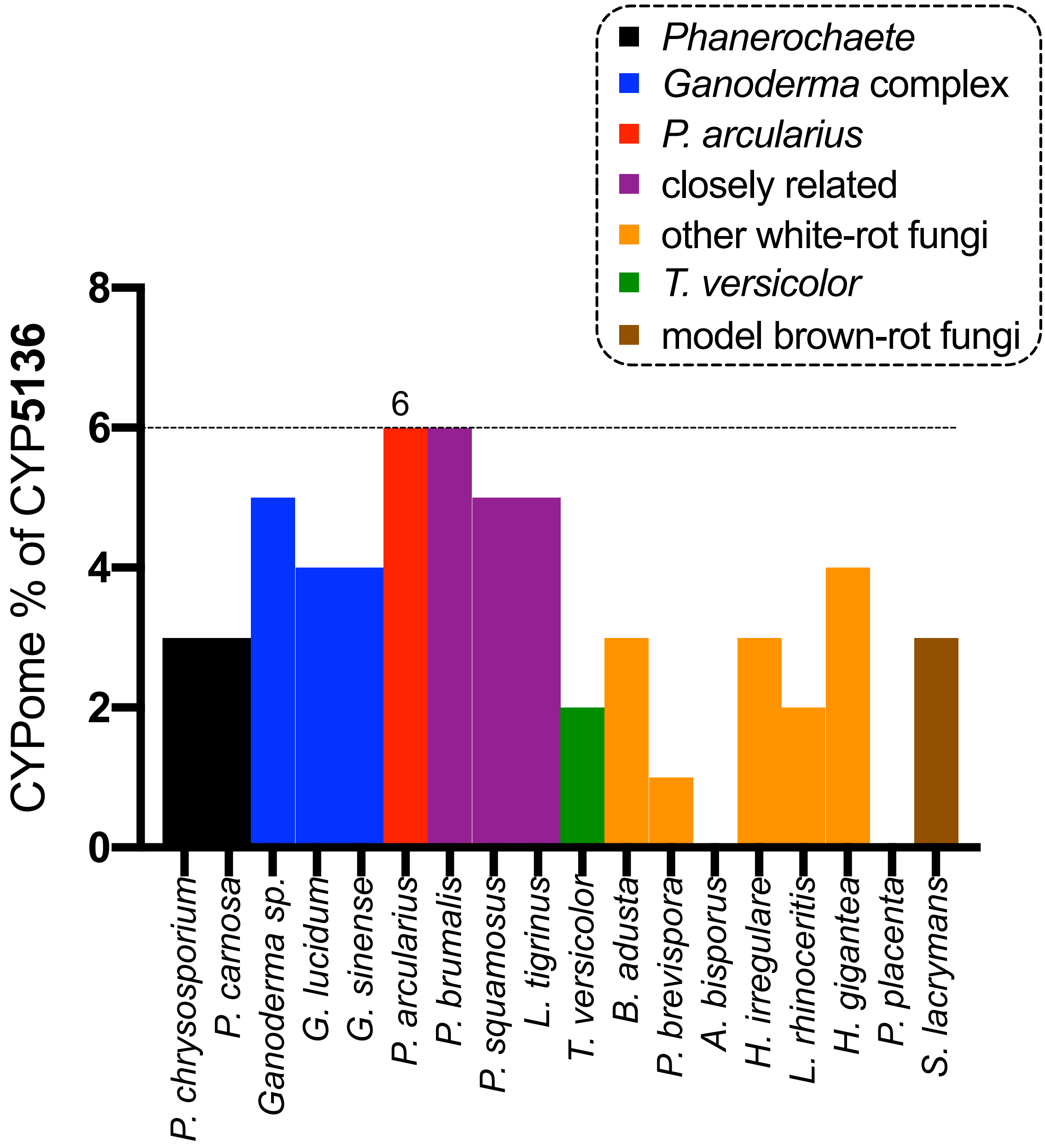

Fig. S5: Comparison of the number of CYP5136s as a percentage of the total number P450s in the genome of *P. arcularius* versus a variety of white- and brown-rot fungi.

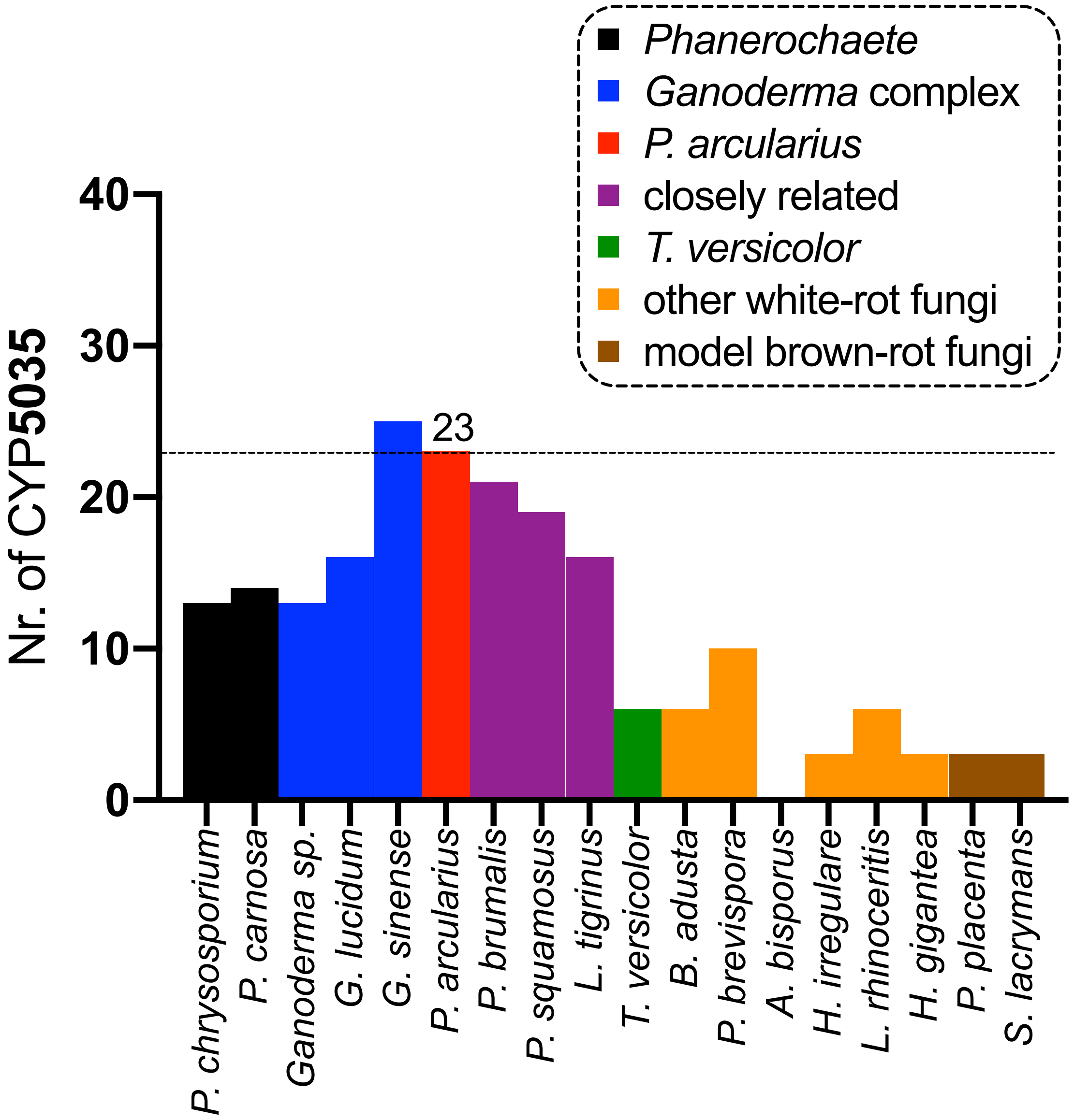

Fig. S6: Comparison of the number of CYP5035 in the genome of *P. arcularius* versus a variety of white- and brown-rot fungi.

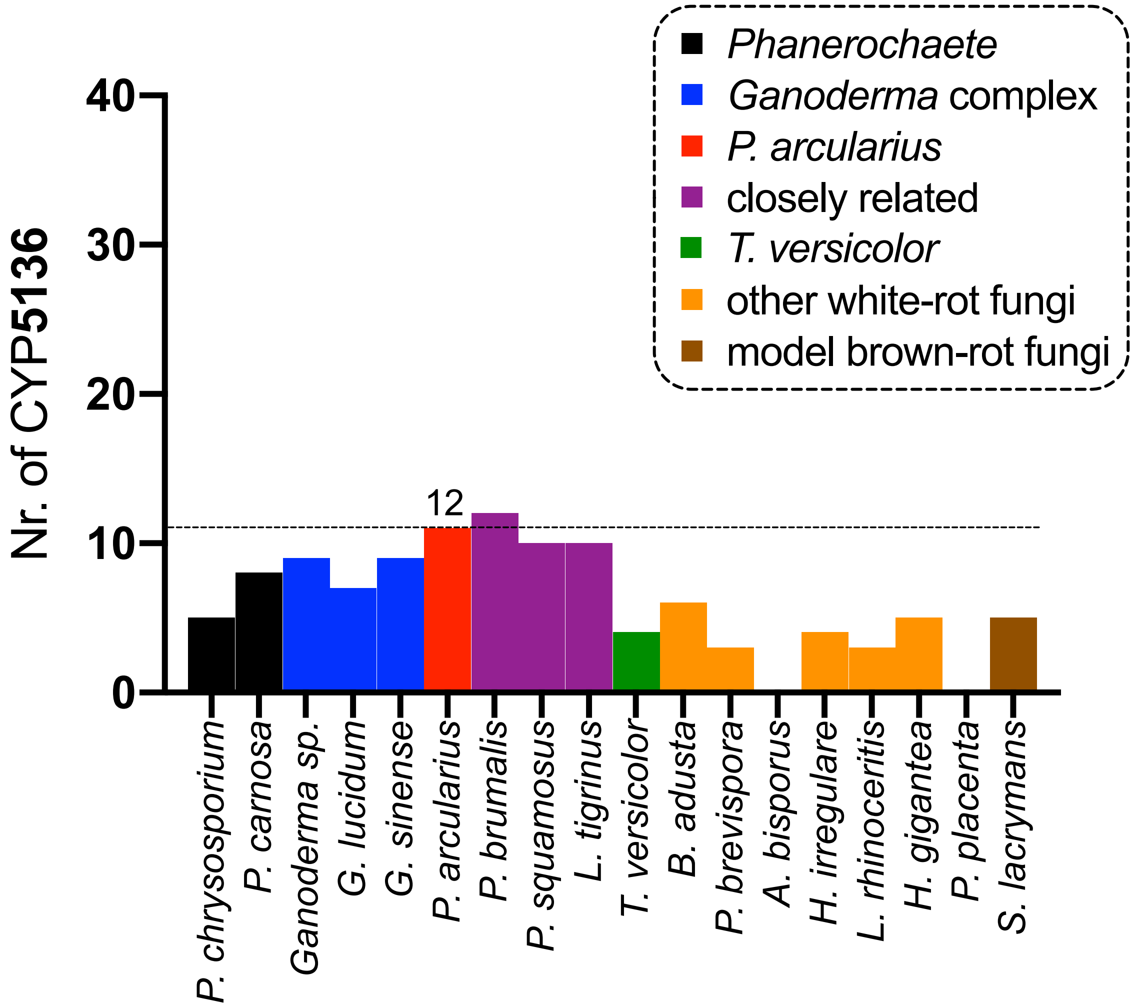

Fig. S7: Comparison of the number of CYP5136 in the genome of *P. arcularius* versus a variety of white- and brown-rot fungi.

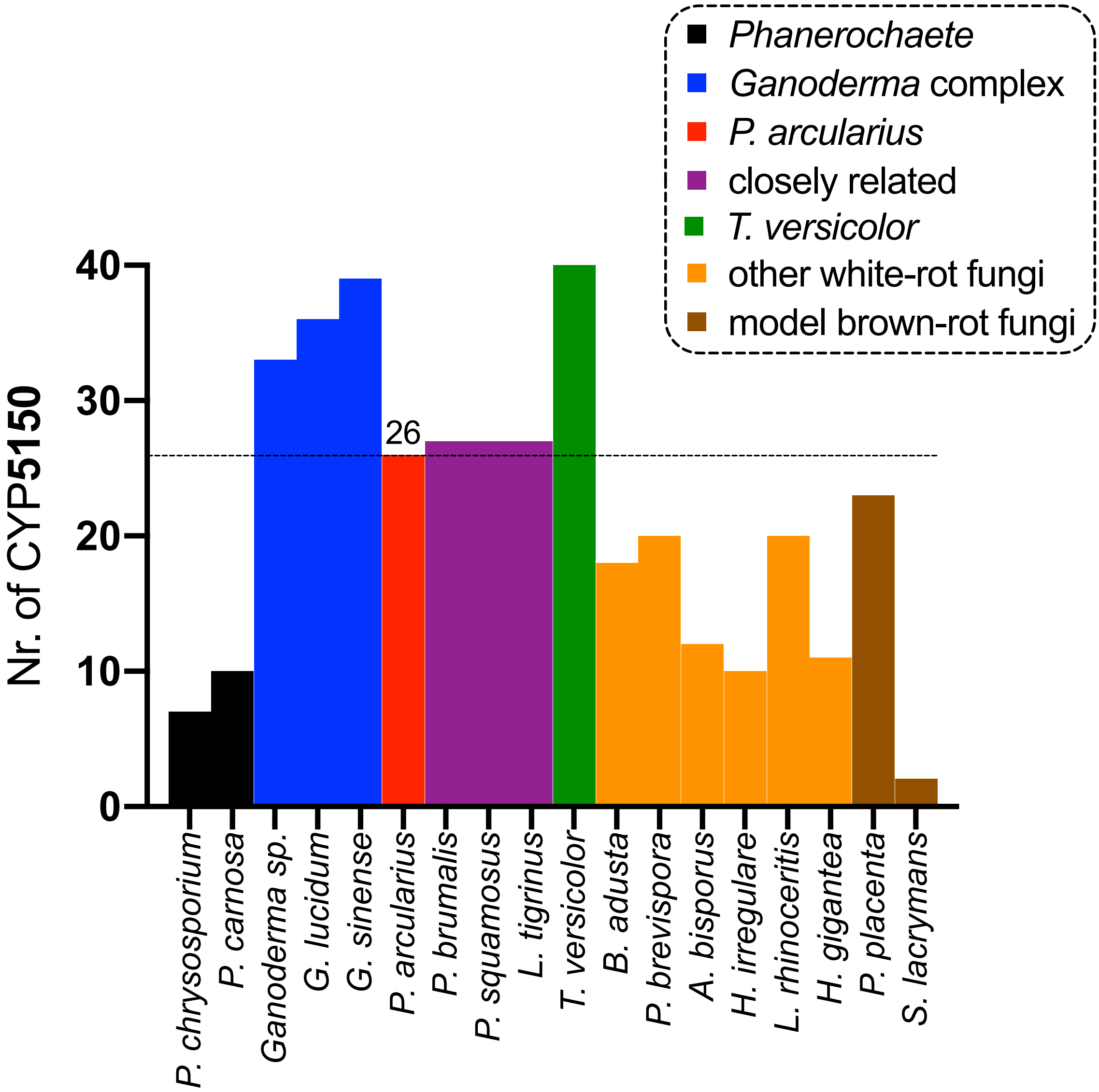

Fig. S8: Comparison of the number of CYP5150 in the genome of *P. arcularius* versus a variety of white- and brown-rot fungi.

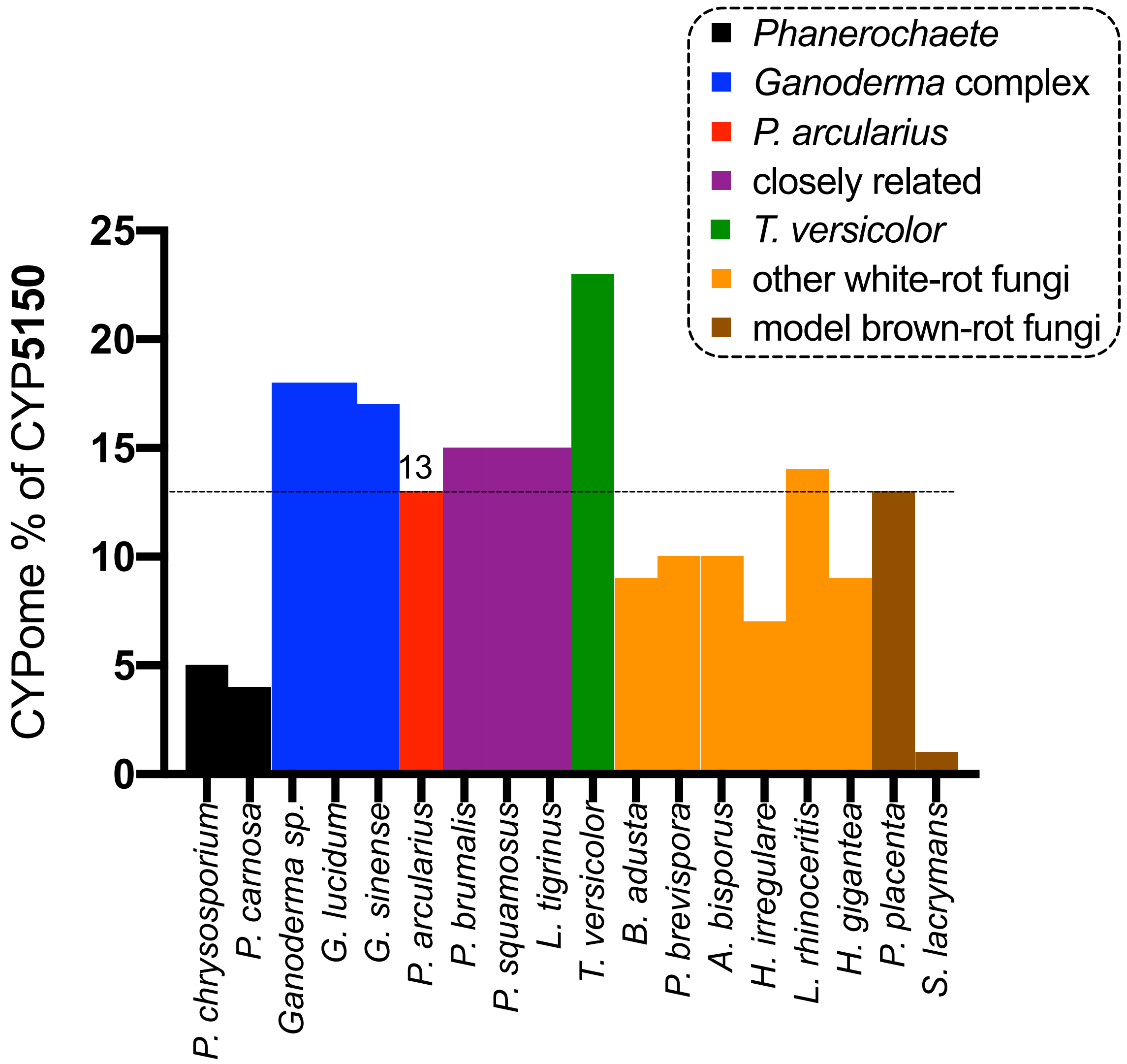

Fig. S9: Comparison of the number of CYP5150s as a percentage of the total number P450s in the genome of *P. arcularius* versus a variety of white- and brown-rot fungi.

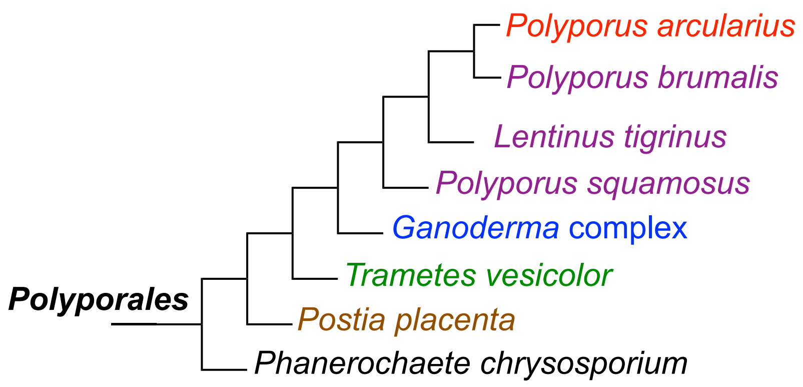

Fig. S10: Rough re-make of the phylogenetic trees drawn in previous studies illustrating the evolutionary distances of different model wood-degrading polypore fungi, and white-rot fungi selected and analysed in this study (Justo and Hibbett 2011; Floudas et al. 2012; Binder et al. 2013).

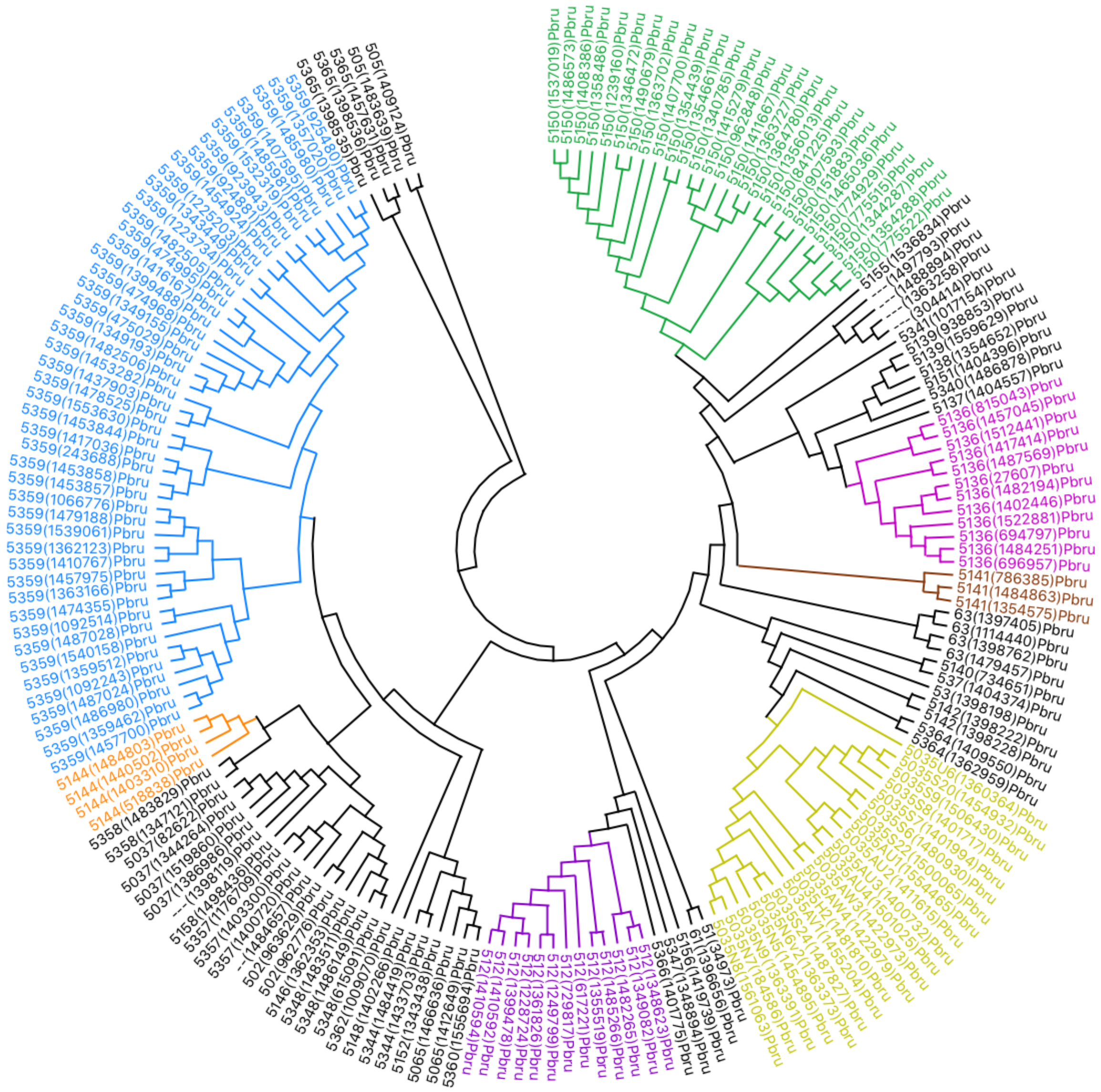

Fig. S11: A minimum evolution tree of the P450ome of *P. brumalis* involving 186 amino acid sequences. CYP512 (purple), CYP5035 (dark yellow), CYP5136 (violet), CYP5141 (brown), CYP5144 (orange), CYP5150 (green) and CYP5359 (blue) have been coloured. The tree was constructed using the close-neighbour-interchange algorithm in MEGA X.

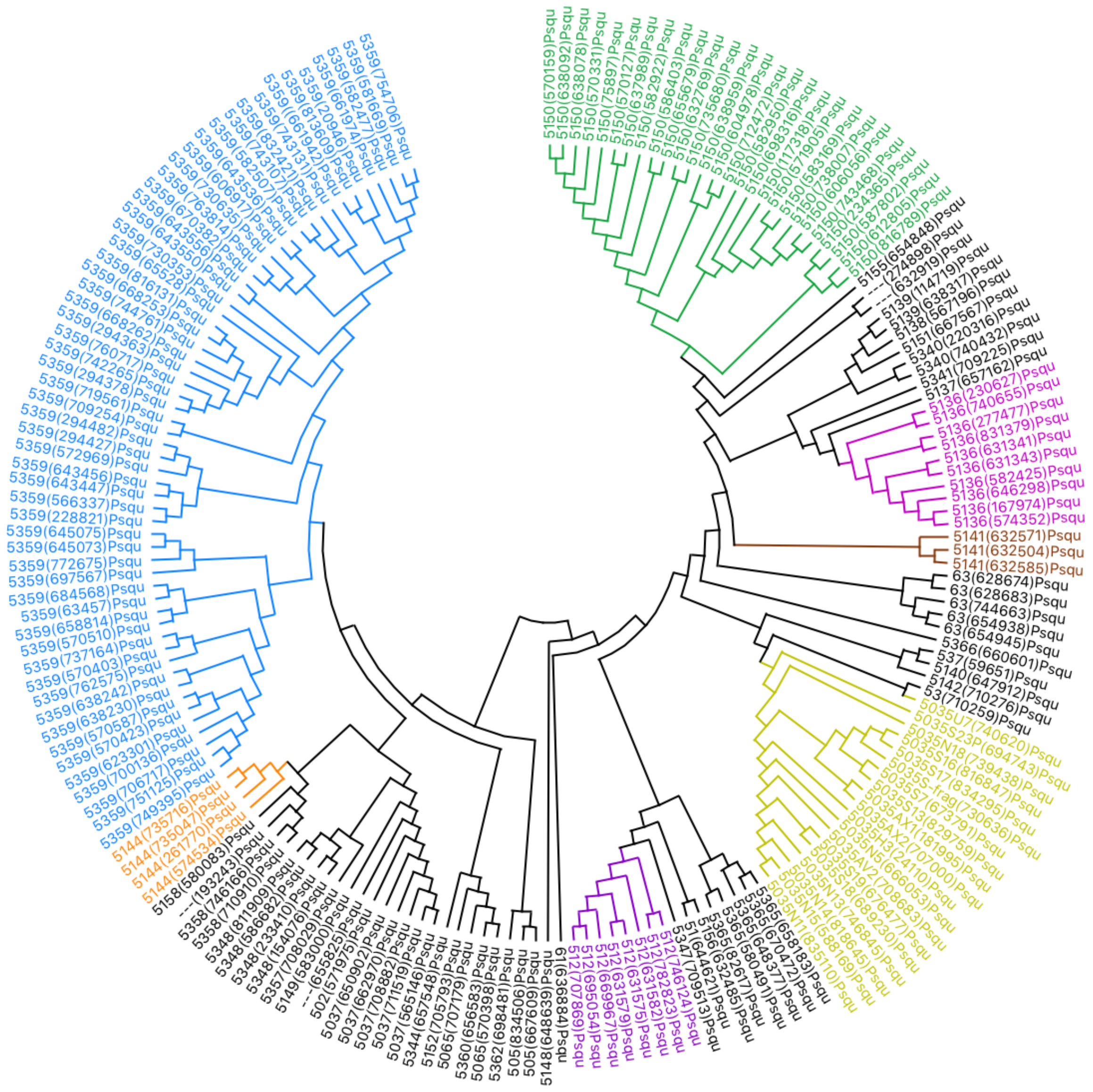

Fig. S12: A minimum evolution tree of the P450ome of *P. squamosus* involving 184 amino acid sequences. CYP512 (purple), CYP5035 (dark yellow), CYP5136 (violet), CYP5141 (brown), CYP5144 (orange), CYP5150 (green) and CYP5359 (blue) have been coloured. The P450 nomenclature of the CYP5035 selected and expressed for a functional screening is shown in red. The tree was constructed using the close-neighbour-interchange algorithm in MEGA X.

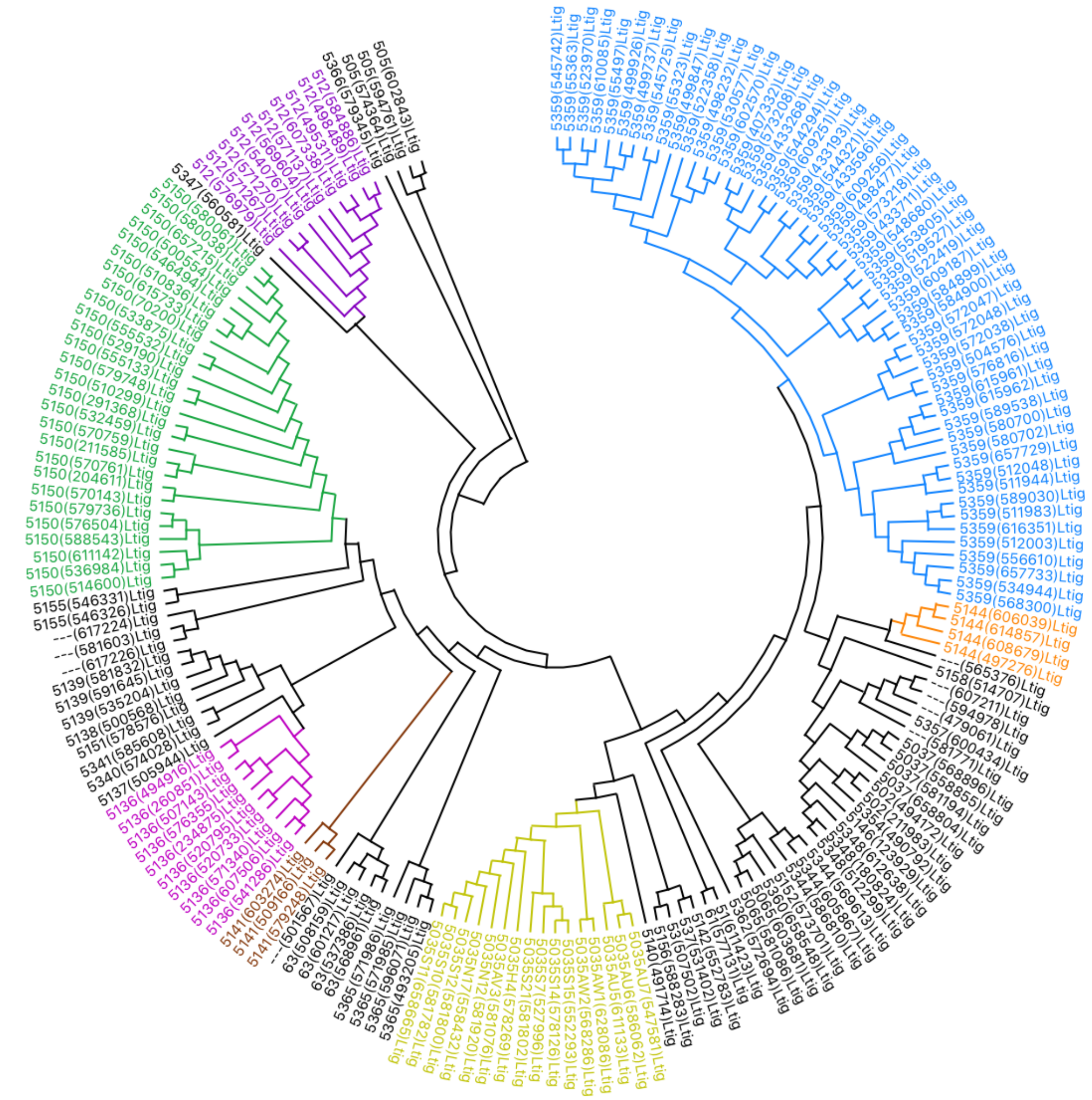

Fig. S13: A minimum evolution tree of the P450ome of *L. tigrinus* involving 184 amino acid sequences. CYP512 (purple), CYP5035 (dark yellow), CYP5136 (violet), CYP5141 (brown), CYP5144 (orange), CYP5150 (green) and CYP5359 (blue) have been coloured. The P450 nomenclature of the CYP5035 selected and expressed for a functional screening is shown in red. The tree was constructed using the close-neighbour-interchange algorithm in MEGA X.

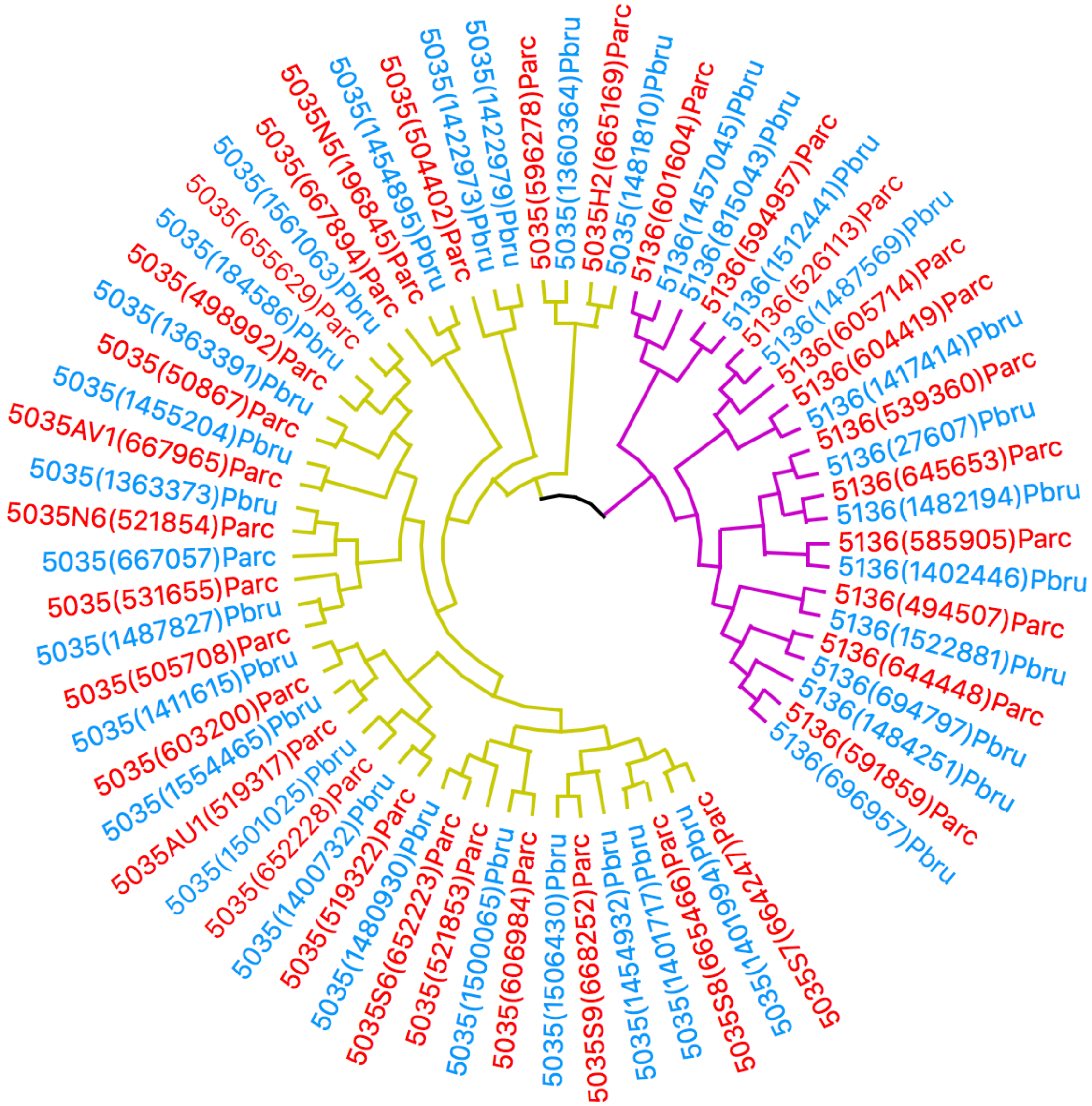

Fig. S14: Displayed is a minimum evolution tree of the CYP5035 and CYP5136 families of *P. arcularius* (Parc; red) and *P. brumalis* (Pbru; blue). Evidence for their close phylogeny is an alternating pattern of red and blue sequences almost throughout the tree. This analysis involved 67 amino acid sequences. The tree was constructed using the close-neighbour-interchange algorithm in MEGA X.

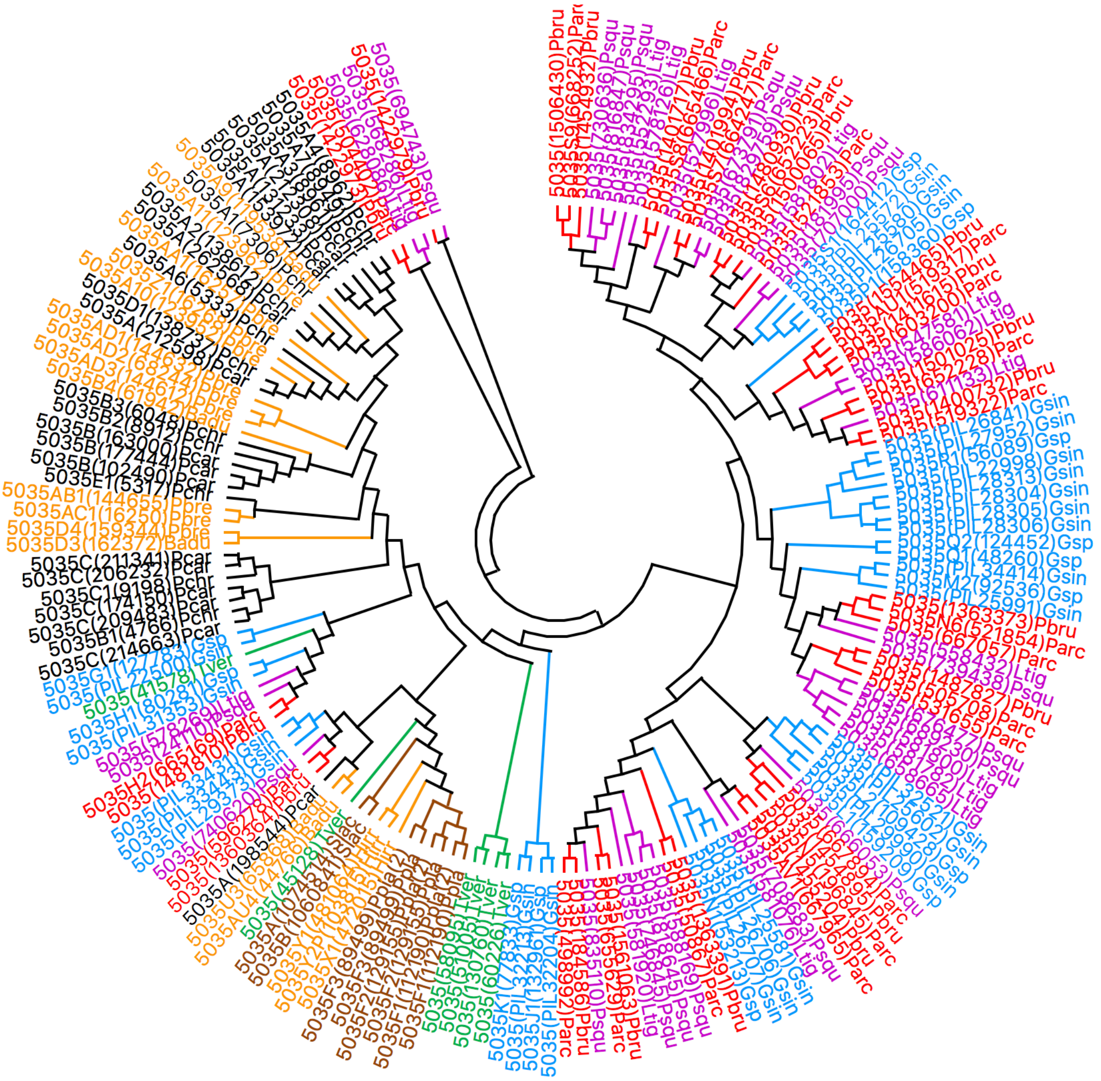

Fig. S15: Displayed is a minimum evolution tree of the CYP5035 family involving 174 amino acid sequences. Phylogeny of CYP5035 enzymes of the fungus *P. arcularius* and *P. brumalis* (Parc and Pbru; red) compared to related species *L. tigrinus* and *P. squamosus* (Ltig and Psqu; violet), and the other model white-rot fungi *Ganoderma* sp. and G. sinense (Gsp and Gsin; blue), *T. versicolor* (Tver; green), *P. chrysosporium* and *P. carnosa* (Pchr and Pcar; black), *B. adusta* and *P. brevispora* and *Heterobasidion irregulare* (Badu and Pbre and Hirr; orange) as well as brown-rot fungi *P. placenta* and *Serpula lacrymans* (Ppla and Slac; brown) in order to get an insight into the evolution of this P450 family. The tree was constructed using the close-neighbour-interchange algorithm in MEGA X.

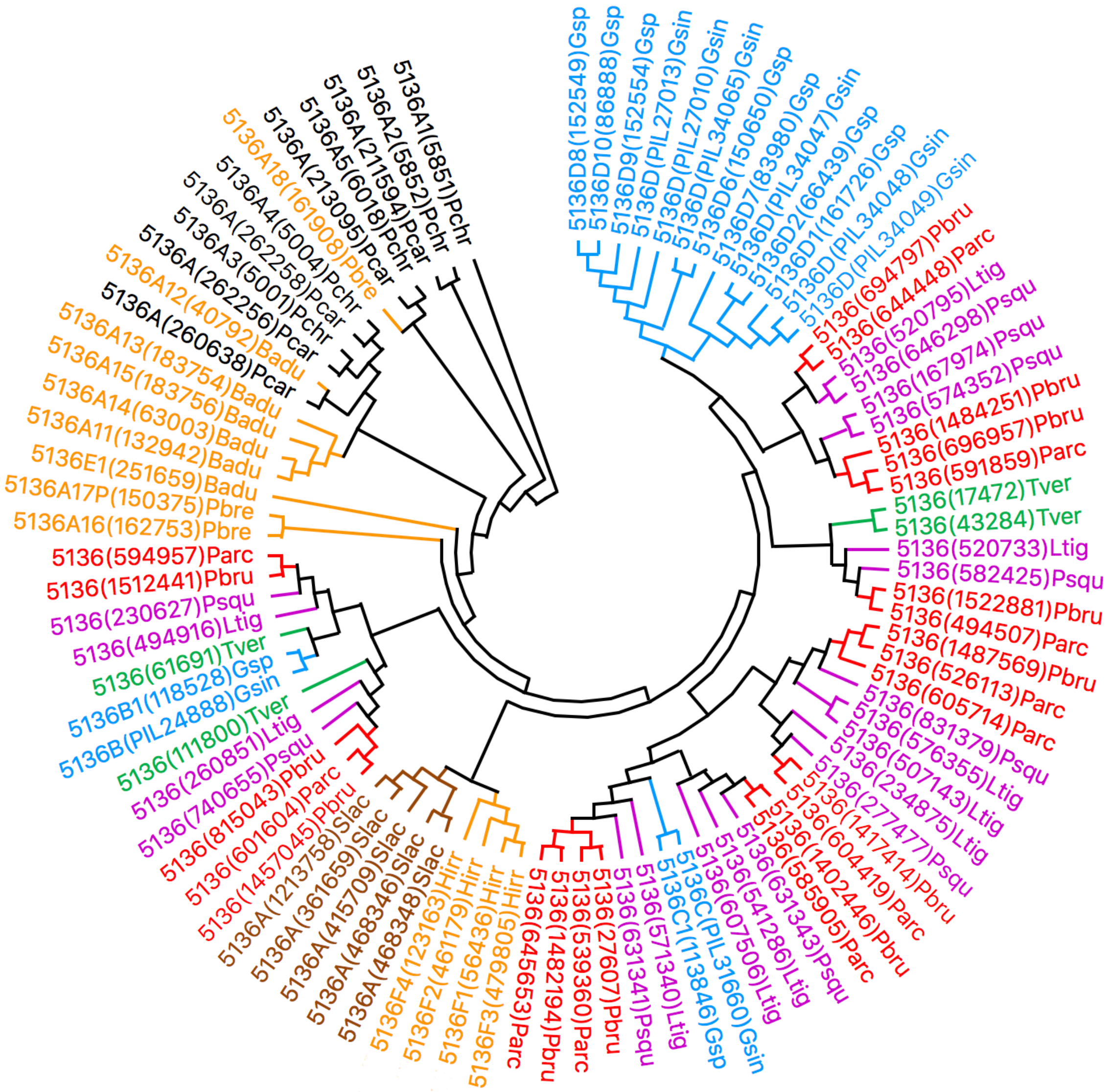

Fig. S16: Displayed is a minimum evolution tree of the CYP5136 family involving 92 amino acid sequences. Phylogeny of CYP5136 enzymes of the fungus *P. arcularius* and *P. brumalis* (Parc and Pbru; red) compared to related species *L. tigrinus* and *P. squamosus* (Ltig and Psqu; violet), and the other model white-rot fungi *Ganoderma* sp. and G. sinense (Gsp and Gsin; blue), *T. versicolor* (Tver; green), *P. chrysosporium* and *P. carnosa* (Pchr and Pcar; black), *B. adusta* and *P. brevispora* and *H. irregulare* (Badu and Pbre and Hirr; orange) as well as brown-rot fungus *Serpula lacrymans* (Slac; brown) in order to get an insight into the evolution of this P450 family. The tree was constructed using the close-neighbour-interchange algorithm in MEGA X.

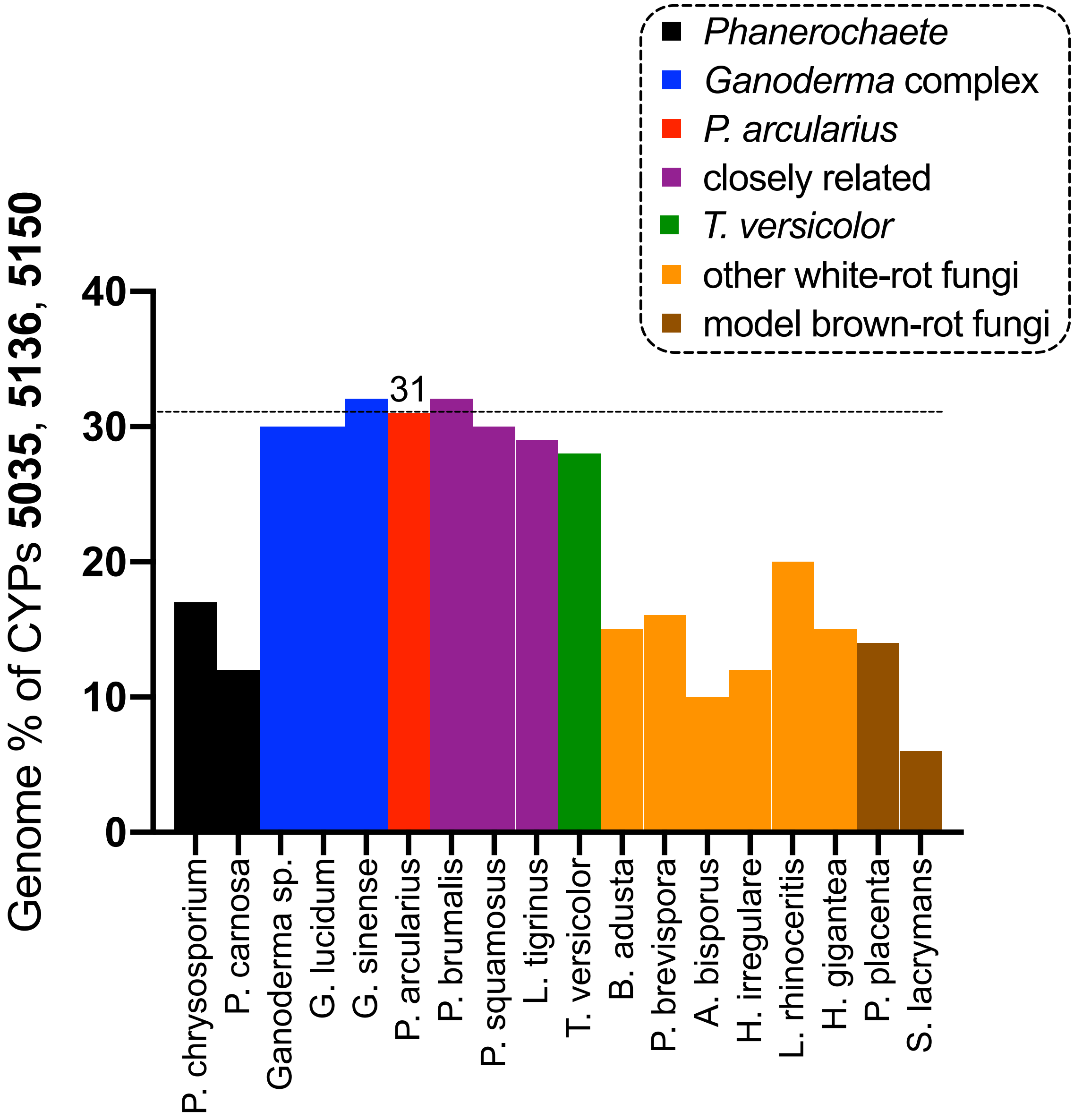

Fig. S17: Comparison of the P450 families 5035, 5136 and 5150 as a percentage of the total number P450s in the genome of *P. arcularius* versus a variety of white- and brown-rot fungi.

Sequences used in this study:

1. **CYP5035N5 of *P. arcularius*** (JGI: 196845; NCBI accession number: TFK85288.1)

MELDGHIYVWSPERLTATYAAVLGLLTHQVFRRHETYCISAHLTLLLAPPLLIALTVSDSWQCIPKTRLLVSYAAYL
STLVLSLVSYRLSPLHPLARYPGPLGCRVSKLWMASLSRAGYQHVYYRDLHKRYGSVVRIGPNELSIREPSAVMALV
GPSGLPKGPHVTGRLLTDKDLPMIGIEDLSTHMTRRRAWNRGFSSAAIAEYEELVGRRAMQLVQRLEEHQGKQVD
IERWFDYFSYDAMCDMTFGGGSELLRDGDENNVWSVLSAGMTAMTFFGHVPWLGVYFGYLPAATRPIKSLLAAC
KGLVEQRMQQGSRTRDLFHYLNHEDQMEQSPPPMRQLVDDGILAIVGGADTVSGALTSVIFCLLTHPETYDKLQVE
VDKYYPPGEDVSSTRWHRDMKYLEAVINETLRVYSPGLGGSQRKVPADGPGVTVGSLYIPPGTALWVHAFSLHRD
PSNFFPFPDDFWPERWLLAPHSPDLLSPEAADAKPTNFVHNEEAYMPFSHGPMNCVGKNFALMEMRIVICALVQ
RFRFRLREDYDRADYDRNFKDYLIASRPNLPVIIELRE

>CYP5035N5

ATGGAGCTGGACGGGCACATCTATGTATGGTCTCCTGAGAGACTCACTGCCACATATGCTGCGGTTCTTGGATTG
CTAACACATCAAGTCTTCCGTCGGCATGAGACTTACTGCATCTCCGCTCACCTTACCCTACTGCTCGCTCCGCCCTT
ACTGATCGCACTTACAGTCTCTGACTCGTGGCAATGCATACCAAAGACAAGACTATTGGTCTCATATGCGGCGTA
TCTCTCTACTCTCGTACTGTCCCTCGTTTCCTACCGCCTCTCTCCATTACATCCTCTCGCGCGCTACCCCGGTCCTC
TCGGTTGCAGGGTGTCCAAGCTGTGGATGGCGTCCCTCTCGCGGGCAGGATACCAACACGTTTACTATAGGGACCT
GCACAAGCGATACGGAAGCGTCGTACGAATTGGTCCGAATGAGCTCTCAATTCGCGAACCTTCGGCGGTGATGGC
CCTGGTAGGCCCGAGCGGACTACCGAAAGGGCCTCATGTCACCGGTAGGCTGCTCACCGACAAGGACCTCCCGATG
ATAGGCATCGAGGACTTGTCTACTCATATGACGCGCCGCCGAGCCTGGAACCGTGGCTTCAGCTCCGCCGCCATAG
CAGAATATGAGGAACTCGTTGGACGACGTGCCATGCAGCTGGTGCAGAGGCTGGAAGAGCACCAGGGCAAGCAAG
TCGACATAGAGCGCTGGTTCGACTACTTCTCGTACGACGCGATGTGCGACATGACGTTCGGCGGCGGGTCTGAACT
TCTACGAGACGGAGACGAGAACAACGTCTGGTCCGTGCTCAGCGCAGGGATGACTGCGATGACGTTCTTCGGTCA
TGTTCCGTGGTTGGGAGTCTACTTTGGTTACCTTCCGGCTGCGACACGTCCGATCAAGTCACTCCTTGCTGCATGC
AAGGGTCTAGTCGAGCAGCGCATGCAGCAAGGTTCGCGGACCCGCGACTTATTCCATTATCTGAATCACGAAGAT
CAGATGGAACAATCTCCACCCCCAATGCGCCAGCTCGTCGACGACGGTATCTTGGCCATTGTTGGCGGAGCGGACA
CCGTATCGGGCGCACTCACAAGCGTGATCTTCTGCCTGCTCACCCACCCGGAGACCTACGACAAGCTTCAAGTCGA
AGTCGACAAGTACTATCCTCCCGGCGAGGACGTGTCCAGCACCAGATGGCATCGAGACATGAAGTACCTGGAGGC
CGTCATAAACGAGACACTGCGTGTATACTCGCCCGGCCTTGGCGGCTCGCAGCGCAAGGTCCCTGCAGACGGTCCT
GGGGTGACAGTGGGCTCGTTGTACATCCCACCCGGAACGGCGTTATGGGTGCACGCGTTCTCGCTACACCGTGACC
CGAGCAACTTCTTTCCCTTCCCAGACGACTTCTGGCCGGAACGCTGGCTCCTCGCCCCCCACTCACCCGACCTCCTC
TCTCCGGAAGCCGCGGATGCGAAGCCAACGAACTTCGTGCACAACGAGGAGGCATACATGCCATTCTCGCACGGGC
CGATGAACTGCGTCGGGAAGAACTTTGCACTAATGGAAATGCGCATCGTCATCTGCGCACTCGTGCAGCGGTTCC
GCTTCCGGTTGCGGGAGGACTACGACCGCGCAGATTACGACCGCAACTTCAAGGACTACTTGATCGCTTCGCGGCC
GAACCTTCCGGTCATCATTGAACTGCGGGAATGA

2. **CYP5035AU1 of *P. arcularius*** (JGI: 519317; NCBI accession number: TFK89225.1)

MLKRAYSSPGAVSVALALITHQVFRRYETYSWFIHGCLLFGPPTLVATFVSDTADTTRSLLQGFFRALPIHLITLSISV
ILYRLSPLHPLAGYPGPLSRKVSMLVPAYLSLSGRKCQYSQALHKQYGDVVRTGPNELSIIDTAAMQHLWNLPRGP
MNVGISLSDKLVPLMGIQDPAEHARRRRPWNRGMSQAAVKEYEHVFADRVHLLVRRLEEQPGKADLAKWIEYLT
YDFMCDMAFGGGSELLREGDKESVFSVMEHGLMVAGALMLVPWLGVYMGYIPGAAKALATLHQTGTRFVLERLE
RGSTTRDLFHYLNNEDLPDTPPPPRQHLVDDGILAVVAGSDTVSSTATSLIYCLITHPDAYADLKVEVDKYYHPGDD
PCNPRHYQDMHYLNAVINETLRIFHPAAAGGQRRVPWDSEPVVAGPYVIPPGTIIMMPQYTIHRDARYFSYPDDF
WPERWLIASGDLRLEDARMPPGKPQLARGEFVHNDAAFIPFGHGPIHCVGKALGVLEMRMLTCALVHKFHFQAP
QGWDAGTYPEQIKEYVTVTRPPLPVVIKPRW

>CYP5035AU1

ATGTTGAAACGAGCGTATTCGAGTCCTGGAGCCGTTTCGGTGGCTCTCGCACTGATTACACACCAAGTCTTTCGCC
GCTACGAGACATACTCATGGTTTATTCACGGGTGTCTTTTGTTCGGACCGCCTACGCTAGTTGCGACCTTCGTCTC
CGACACTGCGGATACTACTCGAAGCCTTCTGCAGGGTTTTTTCAGGGCGCTCCCAATACACCTCATCACGCTGTCG
ATCTCGGTGATACTCTATCGTTTGTCCCCTCTACATCCGCTCGCTGGGTATCCTGGACCTCTTTCGAGGAAGGTGT
CCATGCTGGTTCCGGCATACCTGTCGTTATCAGGACGGAAGTGCCAGTACAGTCAGGCACTCCATAAGCAGTATG
GGGATGTCGTGCGTACAGGGCCGAACGAGTTGTCGATCATCGACACCGCCGCTATGCAGCATCTGTGGAATTTGC
CTAGGGGTCCGATGAACGTTGGAATATCTCTCTCGGACAAGCTCGTCCCCTTGATGGGCATCCAGGACCCGGCGGA
GCACGCACGCCGACGTCGGCCTTGGAATCGAGGAATGAGCCAAGCAGCGGTGAAGGAGTACGAACACGTGTTCGC
AGACCGCGTGCATTTGCTGGTCCGACGTCTCGAAGAGCAGCCGGGCAAAGCGGACCTTGCGAAATGGATTGAGTA
CTTGACATATGACTTCATGTGCGACATGGCGTTCGGAGGCGGCTCAGAGCTGCTGCGAGAGGGAGACAAGGAGAG
TGTCTTTTCGGTCATGGAGCACGGTCTCATGGTGGCCGGTGCACTAATGCTCGTCCCGTGGCTTGGCGTATACATG
GGATACATCCCCGGTGCTGCGAAAGCACTTGCAACTCTCCACCAGACCGGTACTAGATTCGTCCTCGAGCGTTTGG

AGCGCGGGTCGACTACGCGTGATCTCTTCCACTATCTCAACAACGAGGACCTCCCAGATACGCCTCCGCCGCCACG
ACAGCACCTGGTTGACGATGGAATTCTTGCGGTTGTCGCTGGCTCCGACACGGTCTCCAGCACGGCGACGAGCCTG
ATCTACTGCCTCATCACGCACCCGGACGCGTACGCAGATCTGAAGGTCGAAGTGGACAAGTACTACCATCCCGGGG
ACGACCCCTGTAATCCCCGCCATTATCAAGACATGCATTATTTGAACGCTGTGATAAACGAGACTCTTCGTATCTT
CCATCCGGCAGCCGCAGGTGGTCAACGCCGCGTTCCCTGGGATTCAGAACCAGTCGTGGCAGGCCCCTACGTGATC
CCTCCCGGAACGATCATCATGATGCCCCAGTACACCATCCACCGCGACGCGCGATACTTCTCCTACCCGGACGATT
TCTGGCCCGAACGGTGGCTCATCGCCTCCGGCGACCTACGCCTCGAGGACGCGCGCATGCCGCCCGGGAAGCCCCA
GCTTGCACGAGGCGAGTTCGTGCACAACGACGCCGCCTTCATCCCGTTCGGACACGGGCCTATCCACTGCGTCGGG
AAGGCCCTCGGTGTCTTGGAGATGCGCATGCTGACGTGTGCGCTGGTGCACAAGTTCCACTTCCAGGCTCCGCAGG
GGTGGGATGCGGGGACGTATCCGGAGCAGATCAAGGAGTATGTGACGGTGACGAGGCCGCCTCTGCCGGTCGTCA
TCAAGCCGAGGTGGTAG

3. **CYP5035N6 of *P. arcularius*** (JGI: 521854;NCBI accession number: TFK87858.1)

MFRRHETYSIAIHALLLFGAPLLLTVGTASTLTFGVLLSACWTYLVTLILSILLYRLSHLHPLSKYPGPICCRASKLWH
ACVVLKGRQHEYLQALHERYGDVVRIGPNDLSIRDASLIPAILGASGVPKGPNFHGSMLTATNPPMVGIQDTRLHLA
RRRPWARGLALSALKEYHRLVGKRTNQLVHLLENRHGTVVVLGEMFDYFSYDLMCDMAFGGGAELMEEGDPKQ
VWCLLTEGLEAGASFHQMDWLGVYFGHIPAVVKPLQTFLTHGKTLALERMQRGSTRRDLFHYLNEEDLPERTSPP
LQQLVDDGILAIVGGSDTTSSCLTSVFFALLTHPETYVKLQAEVDKYYPAGENPCATKQHRDMTYLHAVINEALRLF
PPVMTLSTRKVPARAPGVHIRSLYIPPGTSISIPPYALQRDPRNFSFPSAFWPERWLIASGQIKLEDAPPPAAASSRT
FEFVHNEVAFMAFSHGPMNCVGKGFALQEIRTVVCALLQRFSFRLGEGWDPREYEATVRDYIVSSRPALPVILERR
TSS

>CYP5035N6

ATGTTTCGTAGACACGAGACGTACTCAATCGCCATCCACGCACTTCTGCTCTTCGGAGCGCCTCTTCTGTTGACCG
TCGGTACGGCCTCGACGCTGACGTTCGGCGTCCTGCTGAGTGCCTGCTGGACTTACCTTGTGACCCTCATCCTCTC
GATCTTGCTCTACCGTCTTTCGCATCTCCATCCACTTTCTAAGTATCCCGGTCCTATCTGCTGCAGGGCGTCGAAG
CTCTGGCACGCCTGCGTCGTGTTGAAAGGACGGCAGCACGAGTATCTACAAGCATTGCACGAGCGATATGGAGAC
GTTGTTCGAATAGGGCCGAACGACTTGTCTATCCGCGACGCCTCTCTCATTCCTGCGATCCTGGGAGCCTCAGGTG
TGCCGAAAGGCCCGAACTTCCATGGGTCGATGTTGACCGCGACCAACCCGCCCATGGTCGGCATACAAGATACTCG
GCTGCACCTGGCACGCCGACGTCCGTGGGCGCGCGGCCTGGCTCTGTCCGCTCTCAAGGAGTACCATCGACTGGTT
GGGAAGAGAACAAACCAGCTCGTGCACCTTCTGGAGAACCGCCATGGAACGGTTGTGGTACTGGGCGAGATGTTC
GACTACTTCAGCTACGACCTCATGTGCGATATGGCATTCGGCGGAGGCGCTGAGCTCATGGAGGAGGGAGATCCG
AAGCAAGTATGGTGCCTTCTTACCGAGGGTCTCGAGGCTGGCGCATCCTTCCACCAGATGGACTGGCTCGGCGTGT
ACTTCGGACACATCCCCGCAGTAGTGAAACCACTTCAAACGTTTCTCACGCATGGCAAGACCCTTGCCCTGGAGAG
GATGCAGCGCGGCTCCACAAGGCGCGACTTGTTCCACTATCTAAACGAAGAGGATCTCCCTGAAAGGACATCGCC
ACCGCTGCAGCAGCTCGTCGATGACGGCATTCTCGCTATCGTGGGCGGTTCTGATACCACCTCTAGCTGCCTCACG
AGCGTCTTCTTTGCCCTTCTCACCCATCCAGAGACATACGTGAAGTTGCAGGCCGAGGTCGACAAGTACTATCCGG
CTGGAGAAAACCCCTGCGCGACCAAGCAGCACCGCGACATGACGTACCTGCATGCAGTGATCAACGAGGCTCTCAG
GCTCTTCCCGCCCGTCATGACCTTGAGTACGCGCAAGGTCCCCGCTCGTGCGCCCGGAGTGCACATCCGGTCCCTA
TACATACCCCCCGGGACTTCGATTTCCATCCCGCCGTACGCACTCCAACGTGACCCACGGAATTTCAGCTTTCCGA
GCGCGTTCTGGCCCGAACGCTGGCTCATCGCGTCCGGCCAGATCAAACTCGAAGACGCTCCTCCGCCTGCCGCGGC
GTCGAGTCGCACTTTCGAGTTTGTGCACAACGAAGTCGCCTTCATGGCGTTCTCGCACGGGCCCATGAACTGTGTC
GGCAAGGGTTTCGCTTTGCAGGAGATCCGGACGGTCGTGTGTGCACTCCTGCAGCGCTTCTCATTCCGGTTGGGGG
AGGGCTGGGATCCGAGGGAGTACGAGGCTACGGTCAGGGACTACATCGTCTCTTCTCGGCCCGCGCTTCCTGTGAT
CCTTGAGCGGAGGACGTCGAGTTGA

4. **CYP5035S6 of *P. arcularius*** (JGI: 652223; NCBI accession number: TFK89222.1)

MRVVGQSAVPTIILALIAHQIFRRHETYYISVHASLLFGVPAGVVLALCWGSHPANALHIGLEVFKTYLITLGISVAVY
RVSPWHPLARFPGPYLRRISHFVSACIYIPGNRSRHFAALHRQYGDVVRTGPNELCIVDPSMIPHILGVPGVPKGPIW
VGGSLSYKTLPLVGIADTEEHMLRRRAWNRGLAPPALREYEVVTASRAKQLVQRLQEQVGEMNLGDWMNRFTYD
FMSDMAFGGGSELLNDGDKDNMWTTISDAMKIAMFLGHVPWLGVYLGYIPPAVSQLKRLLSRGEELAAQRLARGS
TTRDLFHYLNNEDLPDKEPAPRRQLIDDGVLAVVAGSDTTSIVLTSTFYCVLTNPDAYEELQAEIDRHFPQGDDPYI
TQHHRNMPYLQAVINEALRLFPPVPGGTERRVPQHGEPVIAGSLRIPPGTSIFMPPWVLHRDARNFTFPTSFWPER
WLIASGQLSLEKARLPSSIRSPWAQAGDQGAIHPVDFVHNESAYIPFSYGPMNCPGKGLALMELRMVVTAVFQRFK
IRLREGWDPSEYDSGFKDYFNATRPELPVTLERR

>CYP5035S6

ATGAGGGTCGTCGGACAATCCGCCGTGCCGACAATCATCCTAGCTCTGATCGCACACCAGATTTTCCGCCGGCACG
AGACATACTACATCTCGGTTCATGCTTCCTTGCTCTTCGGCGTCCCTGCAGGCGTGGTCCTTGCTCTCTGTTGGGG
GTCTCACCCTGCAAATGCGCTCCACATTGGCCTAGAAGTCTTCAAAACGTATCTGATCACCTTGGGTATCTCCGTG

GCCGTCTACCGCGTCTCTCCCTGGCACCCCCTCGCGCGCTTCCCTGGCCCGTATCTCCGCCGGATATCACACTTCGT
TTCTGCATGCATTTACATTCCGGGAAACCGCTCCCGACACTTCGCTGCACTGCACAGACAATATGGTGACGTGGTG
CGGACAGGTCCAAACGAGCTTTGTATCGTGGATCCGTCTATGATCCCCCATATCCTTGGGGTCCCGGGAGTCCCCA
AAGGCCCAATTTGGGTGGGTGGGTCTCTGTCCTACAAGACCCTCCCGTTGGTTGGCATTGCTGACACGGAGGAGC
ATATGCTGCGAAGACGCGCATGGAATCGCGGTCTTGCTCCTCCAGCACTCAGGGAATACGAGGTCGTCACCGCATC
GAGAGCGAAGCAACTCGTCCAGCGGCTTCAAGAGCAGGTCGGAGAGATGAACCTCGGCGACTGGATGAATCGTTT
TACATATGACTTCATGTCAGACATGGCCTTTGGCGGAGGATCCGAACTTCTCAACGATGGAGACAAGGATAATAT
GTGGACGACTATCAGCGACGCAATGAAAATCGCGATGTTCTTAGGTCACGTTCCTTGGCTAGGTGTCTACCTCGG
CTACATCCCGCCGGCCGTTAGCCAGCTGAAGAGACTGCTGTCTCGTGGAGAAGAGTTGGCGGCGCAGCGACTGGCA
CGAGGGTCCACCACTAGGGATCTATTCCACTATCTTAATAACGAAGACCTTCCAGACAAGGAGCCGGCTCCTCGTC
GTCAGCTCATCGACGATGGGGTACTCGCCGTGGTAGCCGGCTCCGATACCACGTCTATCGTGCTTACATCAACCTT
CTACTGCGTGCTGACGAACCCGGACGCGTACGAAGAACTTCAAGCAGAGATTGACAGGCACTTCCCTCAGGGCGA
CGACCCGTACATCACTCAGCACCATCGCAACATGCCCTATCTGCAAGCGGTGATTAACGAAGCCCTTCGCCTCTTC
CCTCCAGTTCCCGGCGGCACCGAGCGTCGCGTTCCGCAACACGGTGAACCTGTCATTGCGGGATCCTTACGCATTC
CACCCGGTACTTCCATCTTCATGCCCCCATGGGTCCTCCATCGCGATGCGCGCAACTTCACCTTCCCAACCTCGTTC
TGGCCTGAGCGTTGGCTCATTGCGTCCGGGCAACTCTCGCTGGAGAAGGCTCGTCTTCCTTCCTCTATTCGCTCTC
CTTGGGCTCAGGCTGGAGACCAAGGGGCTATCCACCCGGTTGATTTTGTGCACAACGAGAGCGCGTACATCCCGTT
CTCGTACGGCCCGATGAACTGTCCTGGGAAGGGGCTGGCCCTGATGGAGCTGCGCATGGTCGTCACGGCAGTGTTT
CAGCGCTTCAAGATCCGTCTCCGTGAGGGCTGGGACCCGAGCGAGTACGACAGCGGCTTCAAGGACTACTTCAACG
CCACTCGTCCGGAGTTGCCGGTTACACTGGAGCGGCGTTGA

5. **CYP5035S7 of *P. arcularius*** (JGI: 664247; NCBI accession number: TFK85799.1)
MSLREVSPLTIPLAIATHQVFRRYEIYSVSVHACLFLVPPALVAAHISSQSYSPSSIPATFVIALVSYVAAIAASVIVYRL
SPLHPLARYPGPVWRKVSMIGPAILATTGNRAWAFADMHRKYGDIVRSGPNELSIIDPSFIGPLLGASGLPKGPYHV
GASVTPEHVSMAGLQDIPYHLQRRRPWNRGLNPSALKDYQPLIVERLQLLVRRLHEQSGIIDLGLWLKYFAYDFMS
DMAFGGGSELLKDGDKNNIWSIIEEGMVFATILHTLPWLGAYLFKIPGSVKPLLAMQQTTARLAEERFKRGSKTRD
LYYYLSNEDLPDKPPPPLRELADDGVLAVVAGSDTASLTMTSVFYLLLTHPEAYTKLQEEIDTSYHPGEPNAGTKR
HREMPYLHAVINEALRLFPPVPLGTQRQVPHDASPVVFGSVVIPPGTSVYLPTLALHRDPRNFTCADDFWPERWLI
ASGQLRYKEARRPPSLSSLKAADLPDFVHNDVAFTPFSVGPMNCPGKGLAMLEMRMVIVELVKNFVFKLWDGWD
PATYEKEFKDYFTAARPGLPVVLEPRQQL

>CYP5035S7
ATGTCTCTACGTGAAGTATCTCCACTGACGATACCGCTCGCTATTGCTACGCATCAGGTATTCCGACGATATGAGA
TCTACAGTGTCTCCGTCCATGCCTGCCTTTTTCTCGTCCCGCCCGCCCTTGTTGCAGCGCACATCTCCTCGCAATCG
TACTCTCCGTCGTCAATCCCAGCGACGTTCGTAATTGCACTGGTATCCTATGTGGCTGCTATTGCTGCGTCGGTCA
TCGTGTACCGACTTTCTCCGCTCCATCCGCTTGCACGATACCCCGGTCCGGTCTGGCGCAAAGTGTCCATGATCGG
ACCTGCAATACTGGCGACCACTGGCAACCGGGCTTGGGCATTCGCAGACATGCACAGGAAGTATGGGGACATCGT
CCGCAGTGGACCGAATGAGCTGTCTATCATAGATCCCTCATTTATTGGGCCTCTGCTGGGTGCCTCGGGTCTTCCG
AAAGGTCCTTATCACGTCGGCGCATCTGTAACTCCGGAGCACGTATCCATGGCCGGCCTGCAGGATATACCGTACC
ATCTGCAACGCCGACGTCCGTGGAACCGAGGGCTGAACCCAAGCGCTCTGAAGGACTATCAACCTCTGATCGTCGA
GAGACTCCAGCTCCTTGTCCGAAGGCTCCACGAACAGTCCGGTATCATCGACCTCGGACTATGGCTCAAATACTTC
GCGTACGACTTCATGTCTGACATGGCGTTTGGCGGAGGATCAGAGTTGCTGAAAGACGGTGACAAGAACAACATC
TGGTCCATAATCGAGGAAGGCATGGTCTTTGCAACCATACTCCACACTCTGCCTTGGCTGGGCGCGTACCTATTCA
AGATTCCTGGCAGCGTCAAGCCTCTACTTGCAATGCAGCAGACCACCGCTCGACTCGCCGAGGAGCGGTTCAAGCG
AGGCTCCAAAACCCGGGATCTGTATTACTACCTCAGCAATGAAGATCTCCCAGACAAGCCTCCTCCGCCTTTGCGG
GAGCTCGCAGACGACGGCGTCCTGGCAGTCGTTGCGGGCTCAGATACCGCTTCCTTGACCATGACGAGCGTGTTTT
ACCTTCTCCTCACACACCCGGAGGCTTATACGAAGCTGCAAGAAGAAATCGACACCTCATATCATCCTGGTGAGCC
CAACGCGGGGACGAAGCGTCATCGTGAGATGCCCTACTTGCATGCAGTAATAAACGAAGCTCTCCGGCTCTTCCCG
CCGGTCCCTCTGGGTACTCAGCGTCAGGTACCTCACGACGCCTCCCCGGTCGTATTCGGTTCTGTCGTGATCCCTCC
GGGAACCTCCGTGTACCTGCCGACATTGGCACTCCATCGCGATCCTCGCAACTTCACCTGCGCGGACGACTTTTGG
CCAGAGCGCTGGCTCATTGCGTCCGGCCAGCTGCGCTATAAAGAGGCGCGACGTCCGCCCTCCTTGTCTAGCCTGA
AGGCGGCAGACCTTCCCGACTTCGTTCACAACGATGTCGCCTTCACGCCGTTCTCTGTAGGACCTATGAATTGTCC
CGGGAAGGGCTTGGCGATGCTGGAGATGCGCATGGTCATCGTCGAGCTGGTGAAGAACTTCGTGTTCAAGCTGTG
GGACGGATGGGATCCGGCGACGTACGAGAAGGAGTTCAAGGACTACTTTACTGCCGCTCGGCCCGGATTGCCTGT
CGTTCTCGAGCCGAGACAGCAACTGTAA

6. **CYP5035S8 of *P. arcularius*** (JGI: 665466; NCBI accession number: TFK83982.1)
MSLREVSLLTIPFAILAHQVLRRYETYRIYVHACLLLGPPILAAARLTSFRPTPALPPILFNSLVSYLAALVTSVIAYRL
SPFHPLAQYPGPLWRRISMFGPAAMATTGNRHRAFASLHQKYGDVVRTGPNELSIADASFVGPLLGASGLPKGPN
HVGASMSDTKMSMVGIQDIPHHLQRRRPWNRGLSQQALKGYEPLMAERAQLLVQRLTQQSGPVDLGLWLKYFAY

DFMSDVAFGDGSDLLREGDKANIWSIIEDGMVVCTIAHSLPWLGIYLSMIPSAAGPMLAFQENGRRLARERLERGS
KTRDLYHYLCNEDLSDNPPPTLDELADDGTLAIVAGSDTVSVALTSVFYCLLTDTEAHRNVQQEIDRLYPVGEPFSE
TKHHREMHYLQAVINEAMRLFPPIPLGSQRQVPHDAASVVVGSVVIPPGTAIYLPPWVLQRDPRNFTFPDAFWPE
RWLIASGQLHYGDARLPSSAKRGHERPDFVHHEATFIPFSAGPMNCPGKGLAMMEMRSVVIALMKNFGMKLRDG
WNPATFDQEFKDYFTAARPELPVVLEPRLHVETKAYE

>CYP5035S8
ATGTCTCTACGTGAGGTGTCTTTGCTGACGATTCCCTTTGCTATCCTCGCCCATCAGGTGCTCCGCCGGTACGAGA
CGTATCGTATCTACGTCCACGCCTGTCTATTGCTCGGACCGCCCATCCTGGCTGCAGCGCGCCTCACCTCGTTTCG
ACCGACTCCCGCACTTCCCCCCATACTCTTTAACTCTCTGGTATCCTATCTGGCTGCCCTCGTCACTTCCGTCATCG
CTTACCGGCTCTCGCCATTCCACCCCCTTGCACAATATCCCGGGCCTCTCTGGCGCAGAATCTCCATGTTCGGACCC
GCGGCGATGGCGACCACAGGGAACCGTCATAGGGCGTTCGCTAGTTTGCATCAGAAGTACGGAGACGTCGTCCGA
ACCGGTCCGAACGAGCTTTCTATCGCTGATGCGTCGTTCGTAGGGCCGTTACTGGGGGCCTCCGGCCTTCCGAAGG
GTCCCAATCACGTCGGAGCCTCGATGTCCGATACCAAGATGTCGATGGTGGGCATACAAGACATACCCCACCATCT
TCAGCGCCGCCGCCCGTGGAATCGCGGACTCAGTCAACAGGCTCTGAAAGGATACGAGCCGCTCATGGCGGAGCGC
GCCCAGCTTCTCGTACAGCGGCTCACGCAGCAATCAGGACCAGTCGATCTGGGACTCTGGCTGAAGTACTTCGCGT
ACGACTTCATGTCGGATGTGGCGTTCGGTGATGGGTCCGACCTCCTCAGGGAGGGTGACAAGGCCAACATCTGGT
CGATCATCGAGGACGGAATGGTCGTCTGTACGATCGCGCATTCACTCCCCTGGCTGGGCATCTACCTGTCCATGAT
TCCCAGTGCGGCCGGGCCGATGCTTGCCTTCCAGGAGAATGGTAGACGGCTTGCTAGGGAGCGGCTCGAGAGGGG
CTCGAAGACGCGGGACTTGTACCACTACCTCTGTAACGAGGATCTCTCTGACAATCCACCTCCGACGCTGGACGAG
CTCGCGGACGACGGGACCCTGGCCATCGTGGCAGGGTCCGACACCGTTTCGGTGGCGTTAACGAGCGTTTTCTATT
GCCTTCTGACAGACACCGAGGCGCACAGGAATGTGCAACAGGAGATTGATAGGTTATACCCGGTTGGCGAGCCCT
TTTCAGAGACGAAGCATCATCGCGAGATGCATTACCTTCAAGCCGTCATCAACGAAGCTATGCGGCTCTTTCCGCC
CATTCCGCTAGGATCGCAGCGTCAAGTACCTCACGATGCCGCTTCCGTGGTGGTTGGATCTGTCGTCATCCCTCCG
GGTACAGCCATATACTTGCCGCCTTGGGTACTGCAACGCGACCCGCGCAACTTCACCTTCCCCGACGCATTCTGGC
CCGAGCGCTGGCTGATCGCGTCTGGTCAGCTACACTACGGGGATGCGCGACTGCCGTCTTCGGCGAAGAGAGGACA
CGAACGTCCAGACTTCGTCCACCACGAGGCTACGTTCATCCCATTCTCCGCTGGTCCGATGAACTGCCCCGGGAAG
GGCCTTGCGATGATGGAGATGCGCAGCGTCGTCATCGCACTGATGAAGAACTTCGGGATGAAGCTGAGAGACGGG
TGGAATCCGGCGACGTTCGATCAGGAGTTCAAGGACTACTTCACTGCGGCGCGACCTGAGTTGCCTGTGGTGCTTG
AGCCACGACTGCATGTCGAGACAAAGGCCTACGAGTGA

7. **CYP5035AV1 of *P. arcularius*** (JGI: 667965; NCBI accession number: TFK79795.1)
MSTMWYSLAVSALIAHESFKRYETYSIRAHTALLLGPPSLALGFLGSTGSSSRSVLHTLPLAYAAYVGALTVYTILYRI
SPFHPLAQYPGPLGCKVSQWWMACKSWSGYEHLYISELHRKYGDVVRIGPNELSIRDASAISPIMSIAKGPQYVGR
MLSDGIHLPIIGIQDPAEHLRRRRPWNRAFTVPALRGYEETIARRARQLVDALERHNGGQEEVILGKWFNDFAYDF
MCDMAFGGGSELLQERDDSNVWRVLDEGMKVGTLLAHVPWLGVYLSHVPLATGALDVLISHCRMLTTQRVQRG
ATQKDLFHYLNDEDLANSSEKPRAQPPLRQLTDDGCLIVVAGADTTSSALTSLFYCLLTNPETYRRLQDEVDKFYP
RGEDACDTRYHREMRWLNAVICETLRLFPPVPGGSQRQVPHNSAIGVMAGDAFIPPGTSVWAHTWSIHRDPRNFS
RPDAFWPDRWLLASTTLRSSPSSASSSVEADGVRDFVHNEDAWIPFAQGQMNCVGKNLALLEIRMVVCALMQRF
EMRLSEGWDAREYERKFRAYLVATRPEMPVRLRVRCT

>CYP5035AV1
ATGTCGACAATGTGGTACAGCTTGGCCGTATCAGCCTTGATTGCGCACGAGAGTTTCAAGCGCTACGAGACCTAC
TCTATCCGCGCGCACACCGCGCTGCTCCTCGGCCCTCCTAGTCTTGCATTGGGATTCCTCGGCTCCACCGGTTCGAG
TTCACGAAGTGTCCTCCATACGCTGCCCCTCGCGTATGCTGCATACGTCGGCGCTTTAACAGTCTACACTATTCTC
TACCGCATATCGCCCTTTCACCCACTCGCTCAGTATCCCGGTCCGCTGGGATGCAAGGTATCGCAGTGGTGGATGG
CATGCAAATCCTGGTCTGGATACGAACACCTGTACATCAGCGAGCTGCACCGCAAGTACGGGGACGTTGTCCGCAT
CGGCCCAAATGAGCTCTCGATCCGGGACGCGTCTGCGATCAGTCCTATCATGAGCATTGCAAAAGGCCCGCAGTAT
GTCGGACGCATGCTCAGCGATGGGATTCACTTGCCTATAATTGGGATTCAAGACCCTGCCGAACATCTGCGCCGCC
GCCGTCCATGGAACCGCGCGTTTACAGTGCCGGCGTTGAGAGGGTACGAGGAGACGATAGCGCGGAGAGCGCGGC
AGCTGGTCGATGCACTCGAGCGTCATAACGGAGGACAGGAAGAGGTCATACTTGGGAAATGGTTCAATGACTTTG
CCTATGACTTCATGTGTGACATGGCGTTTGGCGGTGGTTCTGAGCTACTGCAGGAACGAGACGACAGTAACGTTT
GGCGGGTCCTGGATGAGGGCATGAAAGTTGGCACGCTTCTCGCACATGTCCCCTGGCTAGGGGTGTATCTGAGCC
ACGTCCCGCTTGCCACCGGGGCACTGGACGTTCTCATCTCGCACTGCCGCATGCTCACCACCCAGCGGGTCCAGCG
GGGCGCTACGCAGAAAGACTTGTTCCATTACCTGAACGACGAAGACCTCGCAAATTCCTCCGAGAAGCCTCGGGC
ACAGCCTCCACTGCGTCAACTCACGGACGACGGCTGCCTCATCGTTGTCGCCGGCGCGGACACCACGTCAAGCGCA
CTTACTAGCCTGTTCTACTGCCTGTTGACGAACCCGGAGACATACAGACGGCTGCAAGACGAGGTGGACAAGTTC
TATCCGCGTGGAGAAGACGCTTGCGACACGAGATACCATCGCGAGATGCGCTGGCTGAACGCGGTAATATGCGAG
ACGTTGCGACTGTTCCCTCCAGTGCCGGGCGGAAGCCAGAGACAAGTGCCGCATAACAGCGCAATAGGCGTCATG
GCAGGTGATGCGTTCATCCCGCCCGGAACTTCGGTTTGGGCACACACTTGGTCCATCCACCGAGACCCGCGCAACT

TCTCCCGCCCTGACGCATTCTGGCCCGACCGGTGGCTGCTGGCAAGTACAACATTGAGAAGCTCCCCCTCATCCGC
ATCAAGTTCTGTCGAGGCGGACGGGGTCCGAGACTTCGTGCACAATGAAGACGCTTGGATTCCGTTCGCGCAGGG
ACAGATGAACTGCGTTGGCAAAAACCTTGCTCTGCTCGAAATTCGGATGGTCGTATGTGCGCTGATGCAGCGCTT
CGAGATGCGCCTGTCTGAGGGATGGGATGCGCGGGAGTATGAGCGGAAGTTCAGGGCGTACCTGGTTGCGACGCG
TCCCGAGATGCCGGTGAGACTGCGGGTGCGTTGTACGTGA

8. **CYP5035S9 of *P. arcularius*** (JGI: 668252; NCBI accession number: TFK79033.1)
MSLREVSPLTLPLALIAHQVFRRHETYRVAVHLSLLLAPPALVAAYVARSQPYSAFLTAFVNALLSYLAALVTSVVV
YRLSSFHPLASYPGPVWRRVSMIGPAAATVSGNRHRTFADMHKQYGDIVRTGPNELSIVDPTFVEPLLGTGGLPKG
PNHIGGNMSEETNLVGIVDIPYHLQRRRPWNRGLKQSALKEYEPLLAERAQLLVQRLGDQSGSADLGLWLKYFGY
DFMCDMAFGGGSELLRNGDKNNVWAIIEEGMVVCTVLHTLPWLGIYLGKIPSVVKPMLLLQENGRQMAKKRLER
GSKTRDLYHYLCNEDLSDRSPPAIAELADDGILTVVAGSDTASMTMTNVFYCLLTHPEAYAKLQAEIDKFYPAGEP
ASETKHHRDMHYLHAVINEALRLYPPVPLGSQRRVPHSGAPVVVGSTVLPPGTVVYLPPWILHRDPRNFSFPDAF
WPERWLITSGQLRHEDAQPPSSAKDATKMDISGLVHNEAAFTPFSIGPMNCPGKGLAMLEMRTVIVSLMKNFSFK
LRDGWDPAKFEEELKDYFLVARPELPVTIERRRIVT

>CYP5035S9
ATGTCTCTTCGTGAAGTATCTCCGCTGACCCTCCCCCTTGCTCTCATTGCACATCAAGTATTCCGCCGACACGAAA
CTTATCGCGTCGCCGTCCATCTCAGCTTGCTGCTTGCTCCTCCTGCGCTCGTTGCGGCGTATGTCGCCCGCTCTCAG
CCGTACAGCGCATTCCTCACGGCGTTCGTGAACGCTCTGCTGTCCTACCTGGCTGCGCTCGTCACCTCCGTCGTCGT
CTACCGGCTCTCCTCGTTCCACCCGCTCGCGAGTTATCCCGGCCCAGTGTGGCGAAGGGTCTCCATGATTGGGCCT
GCGGCTGCGACAGTCTCGGGAAATCGCCACAGGACGTTCGCAGATATGCACAAGCAATACGGCGATATCGTTCGG
ACTGGACCCAACGAACTATCCATAGTGGATCCAACGTTCGTCGAGCCCCTCCTGGGTACTGGCGGTCTCCCGAAGG
GTCCGAATCACATCGGAGGCAATATGTCGGAGGAGACTAATCTGGTCGGCATCGTCGACATACCATACCATCTGC
AGCGTCGCCGTCCCTGGAACCGCGGTCTGAAACAGAGCGCGCTGAAGGAGTACGAACCTCTCCTCGCTGAGAGAGC
ACAGCTTCTCGTGCAGCGTCTCGGTGACCAGTCCGGGTCGGCCGATTTGGGATTGTGGCTGAAGTACTTCGGATAT
GATTTCATGTGTGACATGGCGTTCGGTGGTGGATCCGAGCTGCTCAGGAATGGCGACAAGAACAACGTCTGGGCA
ATCATCGAGGAAGGCATGGTCGTGTGCACGGTCCTGCACACCCTCCCTTGGCTGGGCATCTACCTTGGCAAGATTC
CGAGTGTGGTCAAACCCATGCTGCTGTTGCAGGAGAACGGAAGACAGATGGCTAAAAAGCGTCTCGAGAGAGGGT
CGAAGACTCGGGACTTGTACCACTACCTGTGCAACGAGGATCTCTCCGACAGGAGCCCCCCGGCCATCGCGGAACT
TGCAGACGATGGGATACTTACAGTCGTCGCAGGTTCGGATACCGCCTCAATGACGATGACAAACGTGTTCTACTG
CCTGTTGACGCACCCAGAGGCGTACGCGAAGCTGCAGGCGGAGATCGACAAGTTCTACCCAGCTGGCGAGCCCGCT
TCGGAGACGAAGCATCATCGGGATATGCACTACCTCCACGCTGTGATAAACGAAGCTCTCCGGCTCTATCCACCCG
TCCCGCTGGGCTCTCAGCGCCGGGTACCCCACAGTGGCGCACCCGTGGTAGTCGGCTCAACTGTCCTCCCGCCGGG
TACGGTGGTCTACCTACCGCCCTGGATTCTCCACCGCGACCCGCGCAACTTCAGCTTCCCCGACGCGTTCTGGCCGG
AGCGCTGGTTGATCACGTCTGGCCAACTCCGTCACGAAGACGCGCAACCGCCGTCCTCCGCGAAAGACGCGACGAA
GATGGACATCTCGGGCCTCGTCCACAATGAAGCTGCGTTCACGCCGTTCTCCATAGGCCCGATGAACTGCCCGGGG
AAGGGCCTAGCGATGCTCGAGATGCGCACGGTCATTGTCTCGCTTATGAAGAACTTCAGCTTCAAGCTACGTGAT
GGGTGGGATCCGGCGAAGTTTGAGGAGGAGCTGAAGGATTACTTCCTAGTTGCGCGGCCGGAGTTGCCTGTGACC
ATTGAGAGGAGGCGTATCGTAACATAG

9. **CYP5035H2 of *P. arcularius*** (JGI: 665169; NCBI accession number: TFK84406.1)
MVFQLPAHHALFTVVGSAFIVHLIFKRYEPHRVAVHALLLLAVPSFLSVLLLDRMPAIKALSASFLTFWTALVSSVA
LYRLSPWHPLARYPGPVSLRLSKLSMAWISRGGRRHLYTQELHRRYGDIVRVGPNEVSVSNAAAIHPLMGTSGLHK
GPQWEARTATQSVLPLIAIGDPKEHLRRRKPWNRALNVASLKDFEPFVTHRAEQLVSRLASQKETTNLARWFGW
FTYDLMSDMAFGGGSEMMLNGDDGSVWPLLEIGLLNSDTYGHLPWMADYIRAVPSLGTNMKQMRSFCIQRTEER
IKLGNTSRKDLFYYLNNEDGAEPTPPVPEVTADGTLAIVAGSDTTSSVLSNVFYCLLTHPEAYARLRAEVDSYYPPG
EDALNTKHHADMPYLNAVINETMRLFPPVTDGSQRIVPTGSGGRIIGDSYLPEGTITTVHMYSIQRDARNFAPLPDS
FWPERWLHAAEGARSVIGMKLVHNPTAFFPFSYGPGNCAGKGLALQEMRMVVSAMMQKLELSLAEGFDAVAYE
NEMHDYLILSRPPLPVVVKQRKVCTAEA

>5035H2
ATGGTCTTTCAACTACCCGCTCATCATGCACTATTCACCGTCGTCGGCTCCGCATTTATTGTGCATCTCATATTCA
AACGATATGAACCTCATCGCGTAGCCGTGCATGCTCTGCTGCTGCTTGCCGTACCTTCTTTTCTCAGTGTGCTCTT
GCTTGATCGCATGCCGGCTATCAAGGCACTCTCGGCGTCCTTCCTGACGTTTTGGACCGCACTGGTCTCCTCTGTA
GCCCTCTATCGCCTGTCCCCCTGGCACCCACTGGCCCGCTACCCTGGTCCCGTTTCTCTCAGGTTGTCCAAGTTGTC
CATGGCGTGGATCTCCAGGGGCGGTAGACGTCACCTCTACACGCAGGAGCTTCATCGCCGCTACGGCGATATCGTC
AGAGTTGGCCCGAACGAAGTCTCGGTCAGTAACGCCGCCGCTATCCATCCTCTCATGGGAACATCGGGGTTGCACA
AGGGTCCCCAATGGGAAGCTCGGACTGCCACGCAGAGTGTCCTTCCTCTTATCGCCATCGGCGACCCGAAAGAGCA
CCTGCGTCGCCGCAAGCCATGGAACCGCGCCCTGAATGTGGCCAGCCTGAAGGACTTCGAACCCTTTGTGACACAC

CGCGCAGAGCAGCTCGTCAGCCGCCTCGCATCACAGAAGGAGACGACCAACCTGGCGCGGTGGTTTGGGTGGTTCA
CGTATGACTTGATGAGCGACATGGCGTTCGGCGGCGGGTCTGAAATGATGCTCAACGGAGACGATGGCAGCGTTT
GGCCCTTGCTCGAGATTGGCCTCCTAAATTCAGACACTTATGGTCATCTCCCATGGATGGCAGACTACATCCGCGC
CGTGCCTAGTCTCGGCACGAATATGAAGCAGATGCGGAGCTTCTGCATCCAGCGCACCGAGGAGCGTATCAAATT
AGGTAACACGTCCCGCAAGGACCTGTTCTACTACCTGAACAACGAAGATGGGGCCGAGCCGACTCCGCCTGTCCCT
GAGGTGACAGCTGACGGAACGCTGGCTATCGTCGCGGGCTCAGACACCACGTCAAGCGTGCTCTCGAACGTCTTCT
ATTGCCTCCTCACGCACCCAGAGGCATACGCCCGCCTGCGGGCCGAGGTTGATTCTTACTACCCCCCCGGAGAAGA
CGCCCTCAACACGAAGCATCACGCCGATATGCCTTATCTGAACGCGGTGATCAATGAGACCATGCGTCTCTTCCCA
CCAGTGACGGATGGAAGCCAGCGCATCGTGCCGACAGGTAGCGGCGGAAGGATCATCGGAGACAGCTACCTTCCG
GAGGGCACCATCACGACGGTGCACATGTACAGCATCCAACGGGACGCTCGCAATTTCGCCCCCCTGCCAGACAGCT
TCTGGCCCGAGCGGTGGTTGCATGCTGCAGAGGGCGCGAGGAGCGTGATAGGCATGAAGCTTGTCCACAATCCAA
CGGCGTTCTTCCCGTTCTCATACGGACCCGGGAACTGTGCTGGGAAGGGATTAGCGCTCCAGGAGATGCGCATGGT
CGTGAGTGCCATGATGCAGAAGCTGGAGCTGAGTTTAGCGGAAGGTTTTGACGCTGTCGCCTACGAGAACGAGAT
GCACGACTATCTCATACTGAGTCGGCCGCCACTGCCTGTGGTTGTCAAGCAGCGGAAGGTCTGTACTGCTGAAGCT
TAA

10. **P450 reductase of *P. pastoris*** (NCBI accession number: XP_002494255.1)

MDTLDLSVLIAIALALIAYFSKGSLWGKEDDNSVHGVAGGFQTRDLVEILNSTNKKALVLYGSQTGTSEDYAHKYAR
ELQSKFSIPTLCGDLSEFDFDNLNDIPEQVEGFTFITFFMATYGEGEPTDNAVEFIEFLKNDAEDLSNLKYTVFGLGN
STYEFYNQMGKTTNKRFSELGAQLVGTFGEGDDGQATMDEDFLAWKDSLFDTIKKDLHLEEHEVVYQPGLKVKE
NTALTTSSPNVSVGEPNKAYVLREDENLLQYGPFDHTHPYIAPISSSRELCSETSERNCIHLEFDLSNTNLRYSTGDH
LAVWPSNANEHVESFLKVFNLTDKRSSVFDIEFLDPTVTVHFPFPTTYEAVVRHHLEISGPISRQTLKQFIPYAPDQS
TKQEVIRLSESKDVFHNEVTAKYYNLADLLFKVSKETPWNVPFNFLIETMPNLQHRYYSISSSSLSEKQTIHITAMIE
AFTPTGSDHIVTGVTTNLLWNIQLNQDKSTVKAPVSYDLNGPRNLFSPYKLPVHIRRSTFKLPSNPALPVIMIGPGT
GVAPFRGFIRERCQQVDNGTPNIGQSILYYGCRNSEQDFLYRDEWPTYSKKLGDKFKMYTAFSRENSHKVYVQHRL
LENSREFIELMDQGAFIYVCGDAGKMAKDVNKAIVEILIKEKGLSEEDATESIREFKTSNRYQEDVW

>PpP450red

ATGGATACCTTGGACCTTTCTGTTTTGATTGCTATTGCCTTGGCTTTGATAGCTTACTTTAGCAAAGGATCTCTTT
GGGGCAAAGAGGATGATAACTCCGTCCACGGAGTAGCTGGAGGATTTCAGACCAGAGACCTGGTTGAGATTTTGA
ACTCTACCAACAAGAAGGCACTCGTCCTTTACGGATCACAAACAGGTACATCTGAGGATTATGCTCATAAGTACG
CTCGTGAACTGCAATCCAAGTTCAGCATTCCCACCCTCTGTGGAGACTTGTCAGAGTTTGATTTTGACAATCTGAA
CGATATCCCAGAGCAGGTCGAAGGGTTCACTTTCATCACTTTCTTCATGGCTACGTACGGCGAAGGCGAGCCTACT
GACAACGCCGTAGAGTTTATCGAGTTTCTTAAGAACGACGCCGAGGATCTGTCTAATCTGAAGTATACTGTGTTT
GGGTTGGGTAACTCTACTTACGAGTTCTATAATCAAATGGGTAAGACCACTAACAAGCGTTTCTCAGAACTTGGT
GCTCAATTGGTTGGAACCTTTGGCGAGGGCGATGATGGTCAGGCAACTATGGACGAGGACTTCCTAGCATGGAAA
GATTCCCTGTTTGATACAATCAAGAAGGATTTGCATTTGGAGGAACATGAAGTTGTCTACCAGCCAGGTCTAAAA
GTAAAGGAGAACACAGCGCTTACAACATCTTCTCCTAATGTCTCTGTTGGTGAACCTAATAAGGCTTATGTTCTT
CGTGAAGATGAGAATCTGCTGCAATACGGTCCCTTCGACCACACTCATCCTTACATTGCGCCCATCTCCAGCTCCA
GAGAACTCTGTTCTGAAACTTCGGAGAGAAACTGTATCCATCTAGAATTTGATCTCAGCAACACCAACTTGCGTT
ATTCCACTGGCGATCATTTGGCCGTGTGGCCAAGTAATGCTAATGAGCATGTCGAGAGCTTTTTGAAAGTGTTCA
ACCTCACAGACAAAAGATCTTCTGTGTTTGATATTGAGTTCCTGGACCCTACTGTCACAGTTCACTTTCCGTTTCC
AACCACATACGAAGCAGTTGTTCGTCATCACCTGGAAATTTCTGGACCAATCTCTAGACAGACATTGAAGCAATT
CATTCCTTATGCTCCCGATCAGTCTACTAAACAGGAGGTAATCCGATTATCGGAGAGCAAAGATGTGTTCCACAA
CGAAGTCACAGCAAAATACTATAACCTTGCTGACCTCTTGTTCAAAGTCTCAAAAGAAACTCCATGGAATGTTCC
ATTCAACTTCCTGATCGAGACCATGCCTAATCTGCAGCATCGTTACTATTCTATCTCATCATCTTCATTAAGTGAA
AAGCAAACTATTCATATCACCGCAATGATTGAGGCCTTCACGCCTACTGGAAGTGACCATATAGTGACAGGAGTC
ACCACTAATTTGCTATGGAACATTCAGCTGAATCAGGACAAATCTACTGTTAAGGCGCCTGTCTCCTATGATTTG
AACGGCCCAAGAAATTTGTTTTCTCCATATAAACTTCCAGTTCATATCAGAAGGTCCACTTTCAAACTTCCAAGC
AACCCGGCCCTGCCAGTGATCATGATTGGACCTGGTACTGGGGTAGCTCCATTCAGAGGCTTTATCAGAGAAAGA
TGCCAGCAAGTTGACAATGGAACTCCTAATATTGGTCAATCTATCCTCTATTATGGCTGTAGAAATTCTGAGCAG
GATTTCCTTTACCGTGACGAATGGCCAACATATTCGAAGAAGTTAGGAGATAAGTTCAAGATGTACACTGCGTTC
AGCAGAGAAAATTCACACAAAGTTTATGTTCAGCATAGGTTATTGGAAAACTCAAGGGAATTTATCGAACTGATG
GACCAAGGTGCTTTTATCTACGTATGTGGAGATGCTGGTAAAATGGCTAAGGATGTAAATAAGGCGATTGTTGAA
ATCCTCATTAAAGAGAAAGGATTGTCAGAGGAAGATGCTACAGAAAGTATTAGAGAGTTCAAAACTTCCAACAG
ATATCAGGAAGACGTTTGGTAA

Binder M, Justo A, Riley R, Salamov A, Lopez-Giraldez F, Sjökvist E, Copeland A, Foster B, Sun H, Larsson E, Larsson K-H, Townsend J, Grigoriev I V., Hibbett DS (2013) Phylogenetic and phylogenomic overview of the *Polyporales*. Mycologia 105:1350–1373 . https://doi.org/10.3852/13-003

Floudas D, Binder M, Riley R, Barry K, Blanchette RA, Henrissat B, Martínez AT, Otillar R, Spatafora JW, Yadav JS, Aerts A, Benoit I, Boyd A, Carlson A, Copeland A, Coutinho PM, de Vries RP, Ferreira P, Findley K, Foster B, Gaskell J, Glotzer D, Górecki P, Heitman J, Hesse C, Hori C, Igarashi K, Jurgens JA, Kallen N, Kersten P, Kohler A, Kües U, Kumar TKA, Kuo A, LaButti K, Larrondo LF, Lindquist E, Ling A, Lombard V, Lucas S, Lundell T, Martin R, McLaughlin DJ, Morgenstern I, Morin E, Murat C, Nagy LG, Nolan M, Ohm RA, Patyshakuliyeva A, Rokas A, Ruiz-Dueñas FJ, Sabat G, Salamov A, Samejima M, Schmutz J, Slot JC, St. John F, Stenlid J, Sun H, Sun S, Syed K, Tsang A, Wiebenga A, Young D, Pisabarro A, Eastwood DC, Martin F, Cullen D, Grigoriev I V., Hibbett DS (2012) The Paleozoic Origin of Enzymatic Lignin Decomposition Reconstructed from 31 Fungal Genomes. Science (80-) 336:1715–1719 . https://doi.org/10.1126/science.1221748

Justo A, Hibbett DS (2011) Phylogenetic classification of *Trametes* (Basidiomycota, *Polyporales*) based on a five-marker dataset. Taxon 60:1567–1583 . https://doi.org/10.1002/tax.606003
